# Astrocytes Enhance the Invasion Potential of Glioblastoma Stem-Like Cells

**DOI:** 10.1371/journal.pone.0054752

**Published:** 2013-01-22

**Authors:** Barbara H. Rath, Joshlean M. Fair, Muhammad Jamal, Kevin Camphausen, Philip J. Tofilon

**Affiliations:** Radiation Oncology Branch, National Cancer Institute, Bethesda, Maryland, United States of America; University of Michigan School of Medicine, United States of America

## Abstract

Glioblastomas (GBMs) are characterized as highly invasive; the contribution of GBM stem-like cells (GSCs) to the invasive phenotype, however, has not been completely defined. Towards this end, we have defined the invasion potential of CD133+ GSCs and their differentiated CD133− counterparts grown under standard in vitro conditions and in co-culture with astrocytes. Using a trans-well assay, astrocytes or astrocyte conditioned media in the bottom chamber significantly increased the invasion of GSCs yet had no effect on CD133− cells. In addition, a monolayer invasion assay showed that the GSCs invaded farther into an astrocyte monolayer than their differentiated progeny. Gene expression profiles were generated from two GSC lines grown in trans-well culture with astrocytes in the bottom chamber or directly in contact with astrocyte monolayers. In each co-culture model, genes whose expression was commonly increased in both GSC lines involved cell movement and included a number of genes that have been previously associated with tumor cell invasion. Similar gene expression modifications were not detected in CD133− cells co-cultured under the same conditions with astrocytes. Finally, evaluation of the secretome of astrocytes grown in monolayer identified a number of chemokines and cytokines associated with tumor cell invasion. These data suggest that astrocytes enhance the invasion of CD133+ GSCs and provide additional support for a critical role of brain microenvironment in the regulation of GBM biology.

## Introduction

Glioblastoma (GBM) is the most common form of malignant brain tumor; the current treatment of choice for patients with GBM involves surgery, radiotherapy and temozolomide [1]. Whereas this multimodality treatment approach prolongs survival, the vast majority of patients with GBM succumb to their disease within 1–2 years of diagnosis [1]. Accounting for this poor survival is the presumed intrinsic resistance of GBM cells to standard cytotoxic agents. However, an additional barrier to successful therapy is the invasive propensity of GBM. The capacity of GBM cells to disperse from the primary tumor site and infiltrate the brain parenchyma severely limits the effectiveness of surgery as well as radiotherapy. Thus, developing a strategy that targets the invading cells or restrains their migratory capacity is likely to provide substantial improvements in GBM therapy.

Towards this end, research has been aimed at defining the processes and molecules mediating GBM cell invasion and migration. For the most part these efforts have used long established glioma cell lines. However, in terms of both in vitro and in vivo characteristics, including invasion, these cell lines have little in common with GBM in situ. Data now suggest that GBMs are driven and maintained by a subpopulation of clonogenic cells referred to as glioblastoma stem-like cells (GSCs). These cells have a number of in vitro properties in common with normal neural stem cells including continuous self-renewal; expression of stem cell related genes and the capacity to at least partial differentiate along neuronal and glial pathways [2], [3]. Moreover, when implanted in immuno-deficient mice GSCs form a highly invasive, phenotypically heterogeneous brain tumor [4]. Given the putative significance of GSCs in GBM development and progression, it has been generally assumed that they should also play a major role in determining GBM invasion into normal brain tissue.

The invasive activities of GSCs and non-GSCs have been compared and the mechanism investigated under standard in vitro conditions [5]. However, because invasion reflects an interaction between tumor cells and the surrounding normal tissue, it would seem that to understand the processes mediating GBM invasion it will be necessary to account for the brain microenvironment. Whereas the parenchyma is composed of a variety of phenotypes, astrocytes are the most frequent non-neuronal cell type comprising approximately 50% of the human brain volume [6] and consequently are likely to represent the most frequent point of contact for tumor cells. Astrocytes are a rich source of growth factors, cytokines and other bioactive molecules including proteases and their inhibitors [7], [8]. Moreover, they are intimately involved in the brain response to multiple forms of injury [9], [10]. Finally, astrocytes play a major role in the maintenance and remodeling of the brain extra-cellular matrix [9], [10], [11], [12]. Thus, in an attempt to better simulate the in situ environment and to test the hypothesis that normal brain cells influence GBM behavior, we have used co-culture conditions to define the effects of human astrocytes on GSC and non-GSC invasive activity. The data presented here indicate that under co-culture conditions astrocytes significantly enhance the invasion capacity of GSCs, but not that of non-GSCs. These results are consistent with a role for GSCs in GBM invasion and illustrate the significance of the microenvironment in this process.

## Materials and Methods

### Cell Culture

The neurosphere forming cultures NSC11 [13] (kindly provided by Dr. Frederick Lang, M. D. Anderson Cancer Center) and GBAM1 [14] were isolated from human GBM surgical specimens as described previously [3]. Neurospheres were maintained in stem cell medium (DMEM/F-12, B27 supplement (Invitrogen) and bFGF and EGF (50 ng/ml each, R&D Systems)) and maintained at 37°C in an atmosphere of 5% CO_2_/5% O_2_. CD133+ cells were isolated from GBM neurosphere cultures by FACS as previously described [4], [13] and used as a source for the described experiments. Both CD133^+^ cell cultures met the criteria for tumor stem-like cells [4] ^,^ [13], [14]. For use in an in vitro experiment, CD133^+^ neurosphere cultures (>90% CD133+ cells) were disaggregated [13] and seeded into poly-L-ornithine/laminin (po/ln; Sigma-Aldrich) coated culture dishes. Under these conditions GSCs grow as an adherent monolayer maintaining their CD133 expression [13] and stem-like characteristics [15]. To induce differentiation, CD133+ cells were exposed to DMEM/F-12 and 10% fetal bovine serum (FBS) for 10 days. Differentiation was defined as the loss of CD133 expression, the gain of expression of GFAP and/or β-III tubulin and cell cycle arrest. Human astrocytes (ScienCell) were cultured according to company’s protocol. For conditioned media (CM), astrocytes were seeded into po/ln-coated 6-well tissue culture plates (10^5^/cm^2^); the next day astrocyte cultures were washed twice with stem cell growth media and 3 ml/well of stem cell growth media was added. Conditioned media were harvested after 48 h, sterile filtered and stored for up to one week at 4°C. MRC9 (normal lung fibroblasts) were obtained from American Type Culture Collection (ATCC) in 2010 and maintained in MEM media supplemented with 10% FBS (Invitrogen). ATCC employs short tandem repeat DNA fingerprinting, karyotyping, and cytochrome C oxidase to authenticate cell lines. All cells were cultured less than 3 months after resuscitation.

### Trans-well Invasion Assay

Trans-well inserts (ThermoScientific; 8 µm pore size) were coated on the upper side with po/ln. Single cell suspensions of CD133+ or CD133− cells were stained with the membrane-label dye PKH67 (Sigma-Aldrich) and seeded onto the coated trans-well membrane (10^5^ cells/cm^2^). In those experiments involving specified cell types in the bottom chamber, those cells were seeded at a density of 10^5^ cells/cm^2^ 24 h before insertion of the trans-well membrane. In all experiments invasion was determined 48 h after insertion of the trans-well membrane. Invasion through the membrane was quantified after staining cells on the top and bottom of the membrane as well as on the bottom of the corresponding well with Hoechst 33258 to visualize nuclei of all cells and counting PKH67 stained cells in 6 random fields. Experiments were performed in duplicate with the values presented corresponding to the mean ± SE of at least 3 independent experiments.

### Monolayer-invasion-assay

In this assay CD133+ GSC cells, labeled with a membrane-label dye PKH67, were plated (3.5×10^5^ cells/cm^2^) on po/ln coated glass-cover slips (d:1.2 cm) and allowed to attach over night. For CD133− cells, CD133+ GSCs labeled with PKH67 were seeded (3.5×10^5^ cells/cm^2^) onto po/ln coated glass-cover slips and differentiated as described above. Before addition of the cover slip to the astrocyte-monolayer, membrane labeling was repeated on adherent CD133− cells with PKH67 to ensure adequate labeling. Astrocytes in a single cell suspension were labeled with the PKH26 membrane dye and seeded (10^5^ cells/cm^2^) into po/ln coated tissue culture plates and allowed to adhere over night. Glass-slides carrying CD133+ or CD133− cells- were inverted and placed on top of a confluent astrocyte monolayer, i.e., the GBM cells were in direct contact with astrocytes. After 48 h cultures were evaluated using an inverted fluorescence microscope and Axiovion software (Carl Zeiss). Image analysis was conducted using ImageJ®; green intensity was measured in an area of approximately 2 mm^2^, starting from the edge of the cover slip 1 mm into the astrocyte monolayer and the resulting median green intensity was divided through the measured area.

### Gene Expression Analysis

Microarray gene expression analysis was performed on GBM cells using 2 astrocyte co-culture conditions: indirect, which corresponded to conditions of the trans-well invasion assay, and direct, which corresponded to conditions of the monolayer invasion assay. For microarray analysis of the indirect co-culture, CD133+ or CD133− were seeded (10^5^ cells/cm^2^) onto po/ln coated transwell membranes (0.4 µm pore size) with an astrocyte monolayer (10^5^ cells/cm^2^) in the bottom chamber, or no astrocytes in the bottom chamber (control). After 48 h cells on the transwell membranes cells were lysed in Trizol (Invitrogen). For direct co-culture model, GBM cells were labeled with PKH67 and seeded (10^5^ cells/cm^2^) onto an astrocyte monolayer (10^5^ cells/cm^2^). After 48 h single cell suspensions were generated from the co-cultures, CD133+ or CD133− cells were isolated by FACS according to PKH67staining and RNA isolated. To serve as controls, CD133+ or CD133− cells were seeded on po/ln coated 6-well plates (10^5^ cells/cm^2^) for 48 h. Of note, preliminary experiments indicated that the FACS isolation procedure had no significant effect on gene expression. From all samples RNA was isolated through phenol-chloroform extraction and purified (RNeasy Kit, Qiagen). RNA (100 ng) collected from biological replicates was processed and subjected to microarray analysis on Human Genome U133A 2.0 chips (Affymetrix) according to manufacturer’s instructions. Using Affymetrix Expression Console, Mas5 normalization was performed on all data sets. An expression cutoff of p≤0.05 was implemented to filter all data. Duplicate experiments were averaged and fold-changes were calculated by dividing the expression levels of given co-cultured GBM cells by the corresponding non-co-cultured samples. Probesets that had a fold increase ≥2.0 in co-culture compared to non-co-culture conditions or that were present in co-cultured samples but not in controls were then further analyzed by Ingenuity Pathway Analysis. The data have been deposited in NCBI’s Gene Expression Omnibus and are accessible through GEO Series accession number GSE37120.

### Immunoblotting

GSCs were indirectly co-cultured with astrocytes as described above. After 48 h the GSCs were lysed in 50 mmol/L Tris-HCl (pH 7.5), 150 mmol/L NaCl, 2 mmol/L EDTA, 2 mmol/L EGTA, 25 mmol/L NaF, 25 mmol/L β-glycerophosphate, 0.2% Triton X-100, 0.3% NP-40, and 0.5 mmol/L sodium orthovanadate, supplemented with 1× phosphatase inhibitor cocktails II and III (Sigma-Aldrich), and 1× HALT protease inhibitor cocktail (Thermo Scientific) for 15 minutes on ice. Total protein was quantified with BCA protein assay (Thermo Scientific), separated by SDS-PAGE, transferred to nitrocellulose (Biorad), and probed with the indicated antibodies. Bands were visualized with Pierce ECL Western Blotting Substrate (Thermo Scientific) or IRDye secondary antibodies (LI-COR). Anti- α-Actinin, anti- CCL2 and anti-CD44 were purchased from Cell Signaling Technology. Anti-AGT, anti-HAS2 and anti-AnnexinA2 were obtained from LifeSpan BioScience, Abgent and R&D Systems respectively. Anti–α-Tubulin was obtained from Sigma-Aldrich. Donkey anti-rabbit and sheep-anti-mouse Horseradish Peroxidase conjugated secondary antibodies were purchased from GE Healthcare.

### Cytokine/Chemokine Array

For conditioning of stem cell medium astrocytes were plated in their culture medium at a density of 10^5^ cells/cm^2^ in a 3.5 cm dish, after 24 hours cultures were rinsed twice with DMEM-F12 and medium was replaced with 1 ml of stem cell medium. To serve as a control, 1 ml of stem cell medium was placed in a 3.5 cm dish with no astrocytes and kept in same conditions as the astrocytes at 5% CO_2_/5% O_2_, 37°C. Medium was harvested after 48 h of exposure time from the astrocytes and the control. Conditioned medium from the astrocytes (CM) and control medium were centrifuged and the supernatant was passed through a 0.22 µm filter. Samples were immediately analyzed for cytokine production using the RayBio Human Cytokine Antibody Array G Series 5, testing each sample on two sub-arrays. Remaining sample was stored at −80°C. The antibody array was performed according to manufacturer’s instructions. After blocking the array chip, 100 µl of sample was added per sub-array for incubation. Subsequent washes and biotin-conjugated antibody and fluorescent dye-conjugated streptavidin incubations followed, and fluorescence detection was achieved using a 4000B Axon GenePix laser scanner. Background-deducted signal values were used and normalized against positive controls within the chip. Data are expressed as a ratio of astrocyte conditioned media to control media.

## Results

### Astrocytes Enhance the Invasion Capacity of GSCs

**Figure 1:**
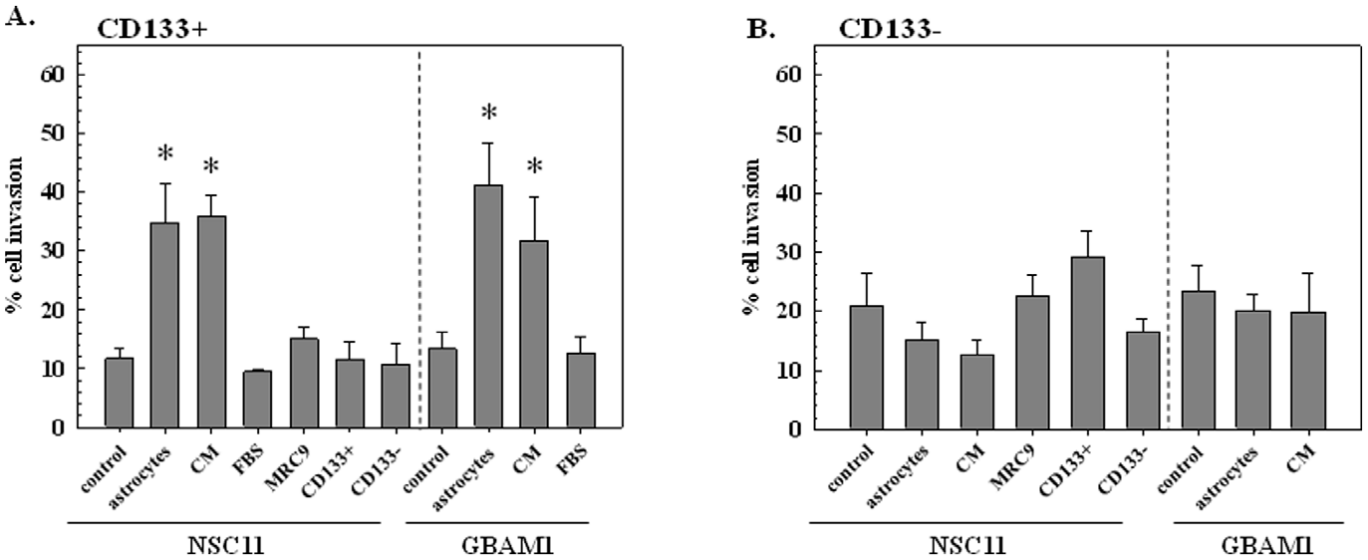
Trans-well invasion assay. A) CD133+ B) CD133− NSC11 or GBAM1 cells were seeded onto trans-well membranes with the specified conditions in the corresponding bottom well, invasion was determined 48 h later (CM: astrocyte-conditioned medium, FBS: fetal bovine serum, MRC9: normal human fibroblasts). Values shown represent the mean ± SE of 3–4 independent experiments. *p<0.05 versus control.

The NSC11 and GBAM1 CD133^+^ GSC cultures used in these studies met the criteria for tumor stem-like cells [4] including self-renewal, differentiation along glial and neuronal pathways, expression of stem cell related genes and formation of brain tumors when implanted in immunodeficient mice [13], [14]. To induce differentiation of the GSC lines, cells were exposed to DMEM/F-12 containing 10% FBS for 10 days, which resulted in the loss of CD133 expression, the gain of expression of GFAP and/or β-III tubulin and cell cycle arrest. Using the NSC11 and GBAM1 GSC lines, the invasion capacity of CD133+ cells and their differentiated progeny (CD133−) was determined using an in vitro trans-well invasion assay. For these studies the top membrane was coated with polyornithine/laminin (po/ln), which approximates the brain extra-cellular matrix [12]. For each cell type the invasion assay was performed using the corresponding growth media, i.e., for CD133+ cells: stem cell medium; CD133− cells: DMEM/F-12 with 10% FBS (see Methods). Under these conditions, the invasion capacity of CD133− cells from both GSC lines was slightly higher, although not statistically significant, than that of CD133+ cells (controls in Figure 1A and B). However, given the different media required for the growth and maintenance of the CD133− and CD133+ phenotypes (e.g. plus and minus 10% FBS, respectively), the direct comparison of their invasion capacity under these control conditions is of questionable validity. In contrast, this model system is amenable to identifying factors or conditions that modify their respective invasion capacities.

To address the potential influence of normal stroma on GBM cell invasion, CD133+ NSC11 cells were seeded onto a po/ln coated trans-well membrane with a confluent monolayer of astrocytes in the bottom well; invasion was determined 48 h later (Figure 1A). Compared to the stem cell media control, the presence of astrocytes in the bottom compartment resulted in a greater than 3-fold increase in invading cells. A similar enhancement in invasion was detected when CD133+ NSC11 cells were plated onto the trans-well membrane in the presence of media conditioned on astrocyte cultures (CM). In contrast, when normal human fibroblasts (MRC9) were grown in the bottom compartment, there was no significant increase in NSC11 CD133+ cell invasion. To determine whether CD133+ invasion could be enhanced by an autocrine effect, NSC11 CD133+ cells as well as their differentiated progeny (CD133−) were plated in the bottom compartment. Neither cell type significantly affected the invasion capacity of NSC11 CD133+ cells. Of interest, addition of FBS, which is often used as an invasion stimulus in this assay [16] and is included in the media used in the analysis of CD133− cell invasion (Figure 1B), had no effect on CD133+ cell invasion (Figure 1A). In contrast to the CD133+ NSC11 cells, astrocytes or their conditioned media had no effect on the invasion capacity of CD133− NSC11 cells (figure 1B). The presence of other cell types (MRC9 fibroblasts, CD133+ or CD133− NSC11 cells) also had no effect on the invasion of CD133− NSC11 cells (Figure 1B).

To determine whether the effects of astrocytes on GSC and non-GSC invasion was unique to NSC11 cells, a similar study was performed using GBAM1 GSCs (CD133+) and their differentiated progeny (CD133−) (Figure 1A, B). As for NSC11, astrocytes as well as their conditioned media significantly enhanced the invasion of GBAM1 CD133+ cells, but had no effect on the CD133− differentiated progeny. It should be noted that during the analysis period (48 h) the GBAM1 as well as NSC11 GSCs maintained their CD133 expression (Figure S1) and did not gain expression of GFAP or β-III tubulin, which indicates that co-culture with astrocytes had no effect on their differentiation status. These results thus suggest that products secreted by astrocytes enhance the invasive potential of GBM GSCs yet have no effect on non-GSCs.

**Figure 2:**
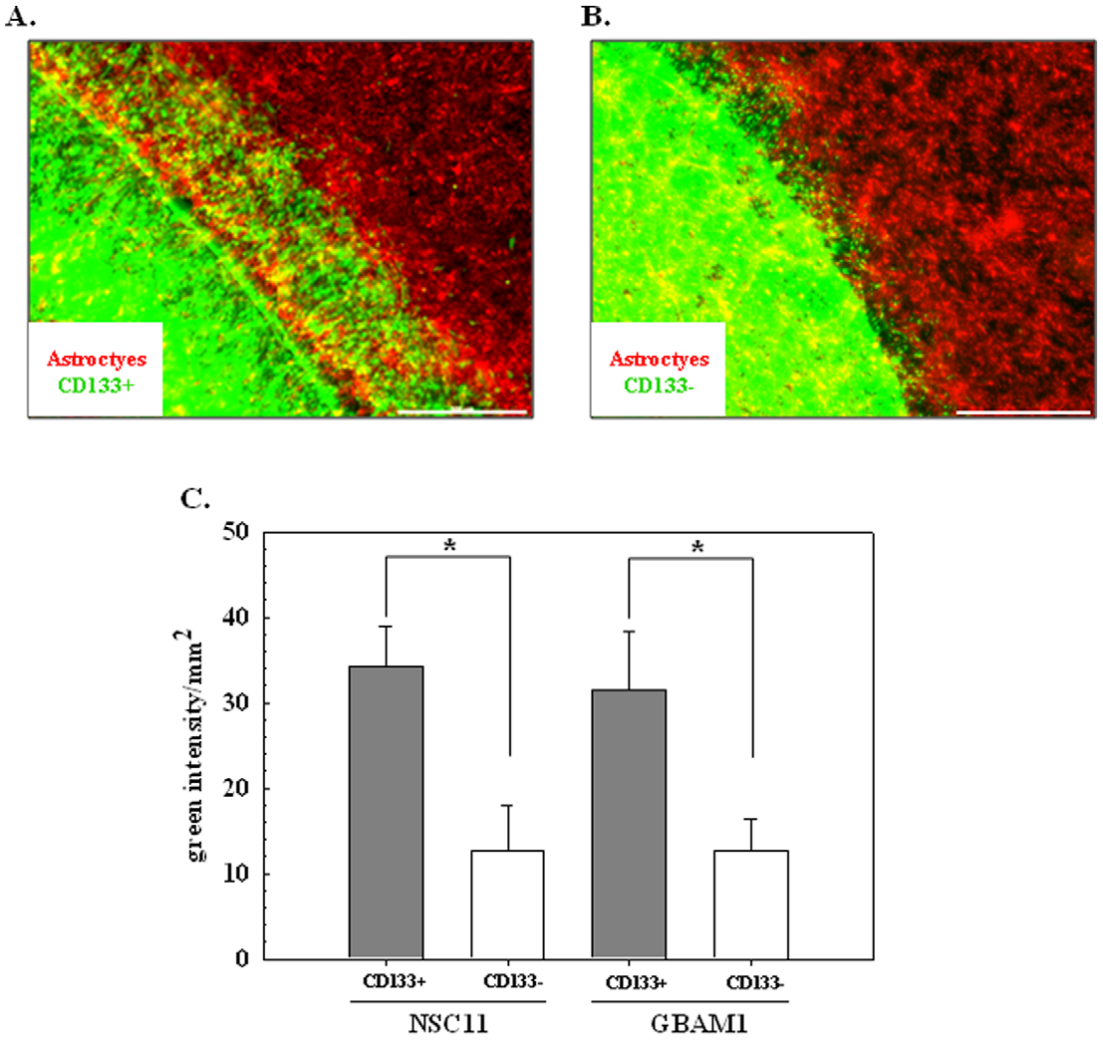
Monolayer-invasion assay. CD133+ GSCs or CD133- cells stained with PKH67 (green) were grown on a cover-slip, inverted and placed on a confluent astrocyte monolayer stained with PKH26 (red) for 48 h. A) Representative image of CD133+ NSC11 cells on astrocyte monolayer. B) Representative image of CD133− differentiated NSC11 cells on astrocyte monolayer (scale bar: 500 µm). C) Invasion of NSC11 and GBAM1 CD133+ and CD133− cells into astrocyte monolayer was defined by image analysis. Values shown represent the mean ± SE of 3–4 independent experiments. *p<0.05.

As an additional assay for investigating the influence of astrocytes on GBM cell invasion, we used a direct co-culture approach. Specifically, a glass cover slip seeded with CD133+ GSCs or their differentiated progeny was placed upside down on an astrocyte monolayer allowing direct contact between GBM cells and astrocytes. After 48 h the number of GBM cells that had migrated away from the cover slip into the astrocyte monolayer was determined and used as an indicator of invasive capacity. Representative micrographs of the co-cultures are shown in Figure 2A and B with green corresponding to NSC11 cells (CD133+ or CD133−, respectively) and red to astrocytes. Similar micrographs were generated for CD133+ and CD133- GBAM1 cells. The extent of invasion into the astrocyte monolayer for each of the GBM cell types was quantified using image analysis with the results shown in Figure 2C. The infiltration of the CD133+ cells from each GBM cell line into the astrocyte monolayer was significantly greater than their differentiated progeny (CD133- cells). Thus, similar to the trans-well invasion assay, these data suggest that astrocytes preferentially enhance the invasive behavior of the GSCs over their differentiated CD133- progeny.

### Astrocytes Modify GSC Gene Expression

**Figure 3:**
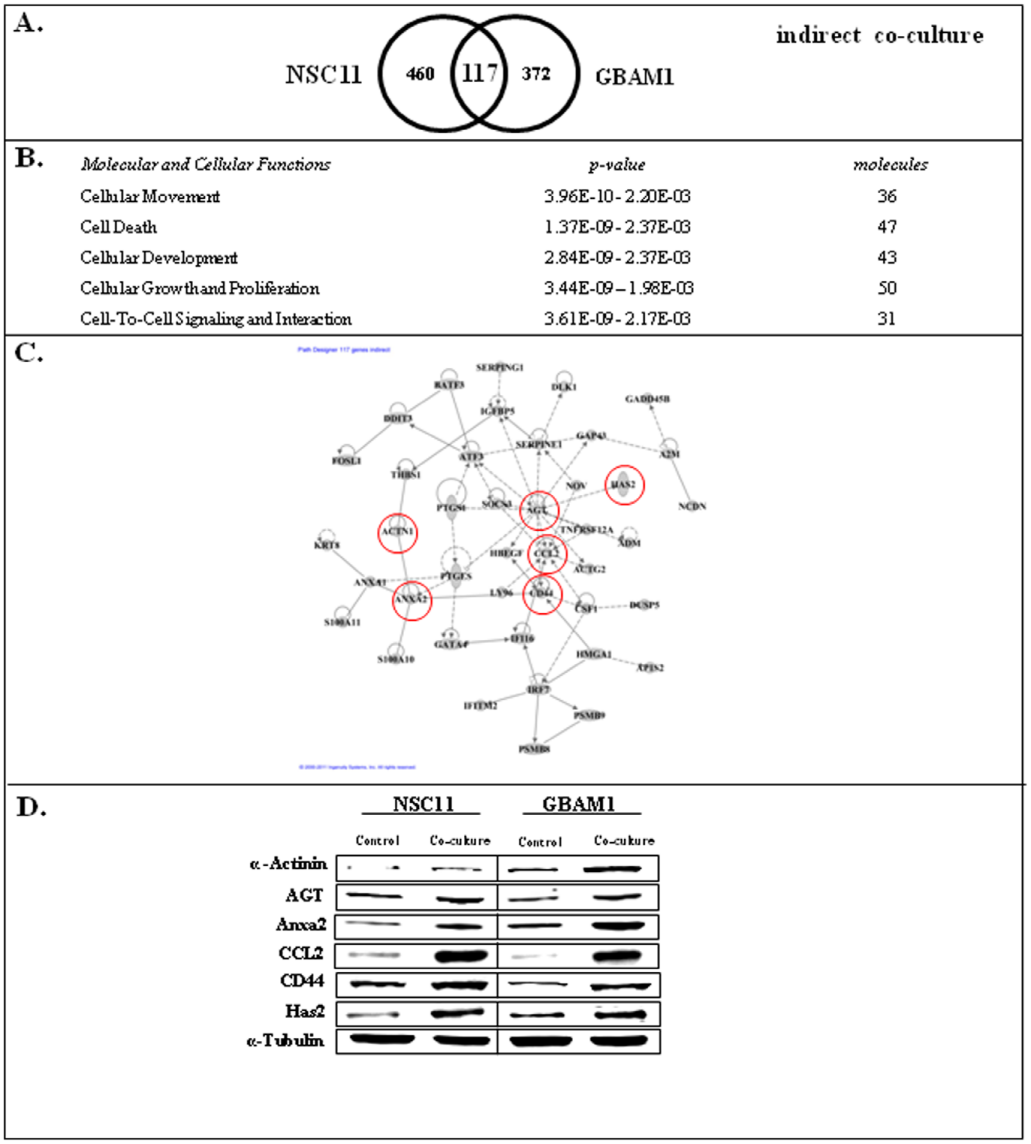
GSC gene expression changes induced by indirect co-culture with astrocytes. A) Venn diagram comparing affected genes in CD133+ NCS11 and GBAM1 cells. B) Commonly increased genes (117) were subjected to IPA and the top five molecular and cellular function categories are shown. C) Interconnecting network formed by 42 of the 117 genes whose expression was commonly increased in NSC11 and GBAM1 CD133+ cells as a result of indirect co-culture with astrocytes. D) Immunoblots generated from GSCs 48 hours after seeding onto po/ln coated trans-well membranes without (control) and with astrocytes (co-culture) in the bottom well. Blots are representative of 2 independent experiments.

To investigate the mechanisms through which astrocytes enhance GSC invasion, we determined the changes in GSC gene expression induced under both co-culture conditions. In these studies, because the aim was to understand the processes that may contribute to cell invasion, we focused on the genes whose expression was increased in GSCs as a result of astrocyte co-culture. The initial analysis addressed CD133+ NSC11 and GBAM1 gene expression under the indirect co-culture conditions of the trans-well invasion assay, simulating the conditions depicted in Figure 1. GSCs were seeded onto po/ln coated trans-well membranes with astrocytes in the corresponding bottom wells. After 48 h the CD133+ cells were collected for microarray analysis of gene expression. The resulting gene expression signatures were then compared to those generated from CD133+ cells grown on the trans-well membrane but without astrocytes in the bottom compartment (control). As shown in Figure 3A there were 117 genes whose expression was commonly increased in the GSC cells as a result of indirect co-culture with astrocytes. Using Ingenuity Pathway Analysis software (Figure 3B), these commonly affected genes were associated with *Cellular Movement* and *Cell-to-Cell Signaling and Interaction*, functions consistent with the influence of astrocytes on GSC cell migration/invasion. Moreover, of the 117 genes, 42 were distributed to an interconnected network (Figure 3C; bold genes in Table S1). Of the hub genes within the network *CCL2*, *CD44*, *ANXA1* and *ANXA2* have been linked to cell migration/invasion [17], [18], [19], [20]. In addition, there are a number of other non-hub genes that have been associated with cell migration/invasion such as *HAS2* [*21*] and ACTN1 [22] (Table S1).

To determine whether the changes in gene expression detected by this microarray analysis corresponded to changes in protein expression, immunoblot analysis was performed on GSCs grown on po/ln coated trans-well membranes with and without astrocytes in the corresponding bottom wells. The proteins evaluated were those previously established to play a role in the invasion and migration of cancer cells: ACTN1, AGT, ANXA2, CCL2, CD44, HAS2 [17], [18], [20], [22], [23], [24] and denoted by the red circles in Figure 3C. As show in Figure 3D, the levels of each these proteins in the GSCs was increased as a result of co-culture with astrocytes, which is consistent with the microarray-based gene expression data.

**Figure 4:**
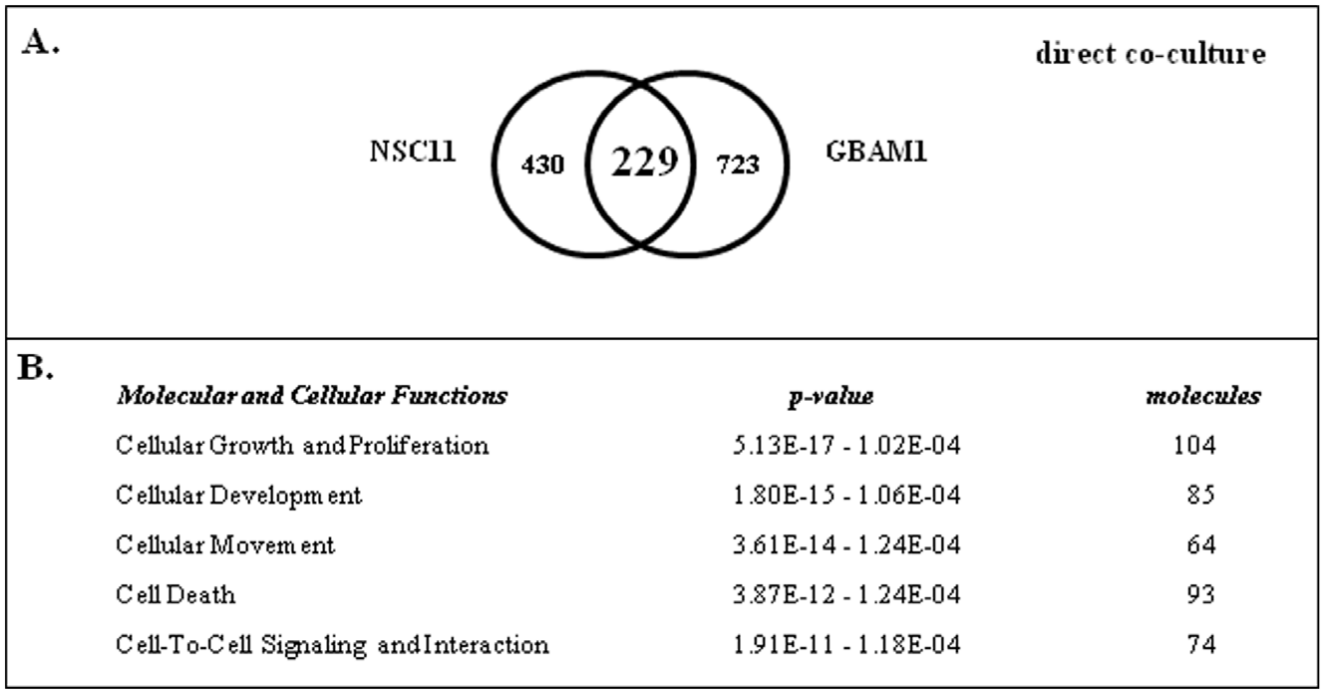
GSC gene expression changes induced by direct co-culture with astrocytes. A) Venn diagram comparing affected genes in CD133+ NCS11 and GBAM1 cells. B) Commonly increased genes (229) were subjected to IPA; the top five molecular and cellular function categories are shown.

**Figure 5:**
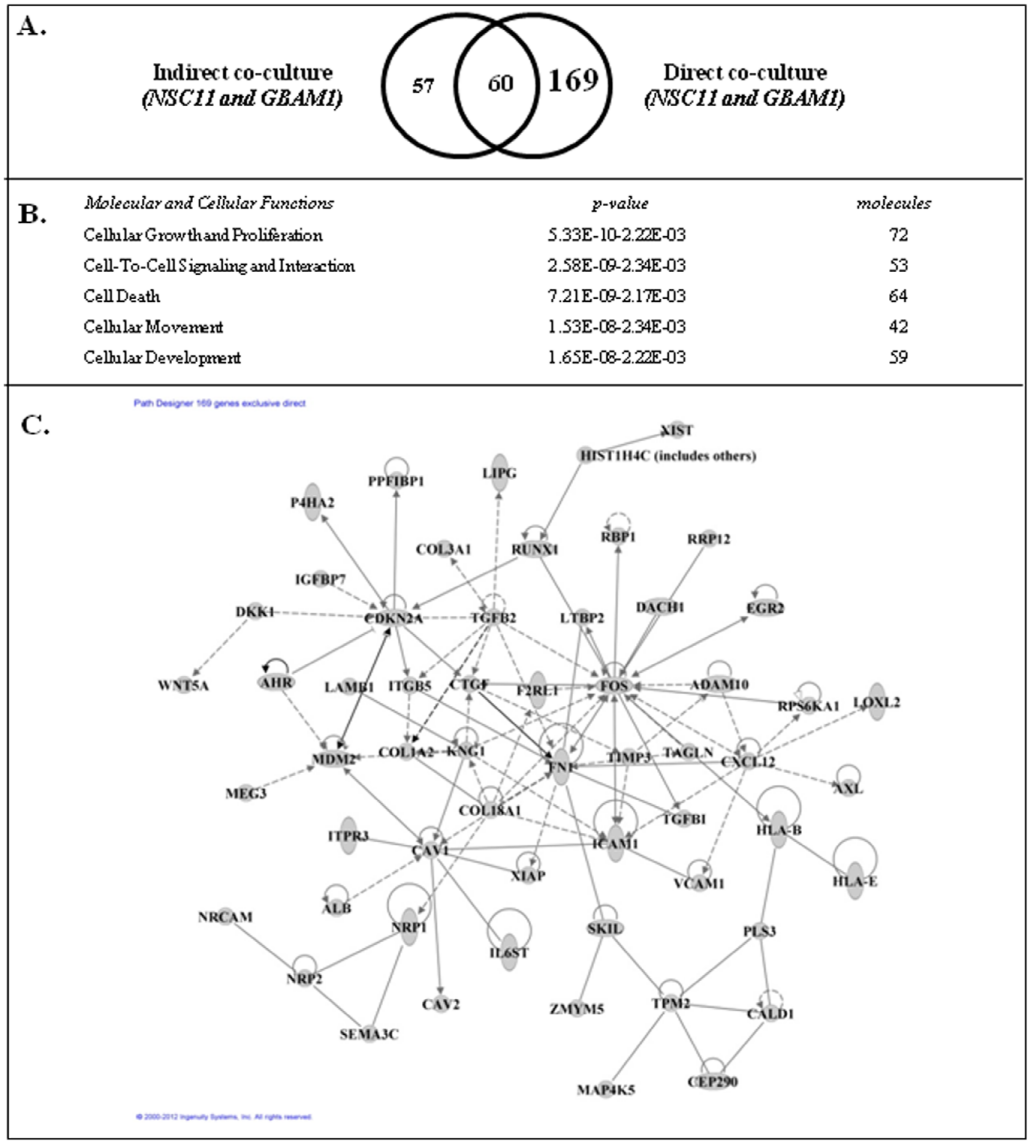
Comparison of GSC gene expression changes induced by indirect and direct co-culture with astrocytes. A) Venn diagram comparing genes whose expression was increased in both CD133+ NCS11 and GBAM1 cells after indirect and direct co-culture with astrocytes. B) Genes commonly affected only in direct co-culture with astrocytes (169) were subjected to IPA; the top five molecular and cellular function categories are shown. C) Interconnecting network formed by 58 of the 169 genes whose expression was increased in both NSC11 and GBAM1 CD133+ cells only as a result of direct co-culture with astrocytes.

We also defined the gene expression changes induced when GSCs were grown in direct contact with astrocytes, mimicking the conditions used in Figure 2. For this analysis GSCs were seeded onto an astrocyte monolayer. After 48 h, a single cell suspension was generated from the co-culture; the GSCs were isolated using FACS and subjected to microarray analysis. The resulting gene expression signatures were then compared to those generated from individual cultures of CD133+ GSCs. As shown in Figure 4A, direct contact with astrocytes resulted in 229 genes commonly increased in NSC11 and GBAM1 GSCs. Using IPA the 229 genes (Figure 4B) were associated with *Cellular Movement* and *Cell-to-Cell Signaling and Interaction*, functions consistent with cell invasion/migration. Of the 229 genes 107 were distributed to an interconnected network, which because of the number of genes involved is shown in Figure S2. The complete list of genes includes such invasion/migration associated genes as *ADAM10* [*25*], *HAS2* [*21*], *IL6ST* [*26*], *VCAM1* [*27*] (Table S2). The GSC genes induced by the direct co-culture with astrocytes may reflect genes induced by secreted astrocyte products, similar to those in the indirect co-culture, and/or those induced as a result of direct cell-cell contact with astrocytes. To gain insight into the genes selectively induced by direct contact, we compared the genes commonly increased in the GSCs by both the indirect (117) and direct (229) co-culture with astrocytes. As shown in Figure 5A, 60 genes were commonly increased as a result of exposure of GSCs to both co-culture conditions. However, 169 genes (Table S3) were unique to the direct co-culture and would thus appear to reflect those whose increased expression required cell to cell contact between the GSCs and astrocytes. These genes also distributed to molecular and cellular functions associated with cell invasion/migration such as *Cellular Movement* and *Cell-to-Cell Signaling and Interaction* (Figure 5B). In addition, of the 169 genes exclusive to the direct co-culture model 58 could be assigned to an interacting network (Figure 5C; bold genes Table S3) including such genes *CTGF* [*28*], *TGFB-2* [*29*] and *CXCL12* [*30*], which have been associated with invasion/migration. These results thus suggest that direct contact and astrocyte secreted products act to enhance the invasive capacity of GSCs.

### Astrocytes Modify CD133− Cell Gene Expression

To determine whether the astrocyte induced expression of invasion/migration related genes was unique to CD133+ cells, the same experiments were performed using their CD133− differentiated progeny. The number of genes that were commonly increased in CD133− NSC11 and GBAM1 cells by direct or indirect co-culture with astrocytes was 1033 (Figure S3). These genes were subjected to IPA; none of the top 5 networks could be directly associated with cell invasion/migration. In contrast, the same analysis performed on the total number of genes whose expression was commonly increased in CD133+ NSC11 and GBAM1 cells by both co-culture conditions (286 genes) showed that the top 5 networks included *Cellular Movement* and *Cell-Cell Signaling and Interaction*. These results are consistent with an astrocyte-mediated selective enhancement of CD133+ cell invasion potential as compared to CD133− GBM cells.

### Astrocytes Secrete Proteins Associated with Cell Invasion

**Figure 6:**
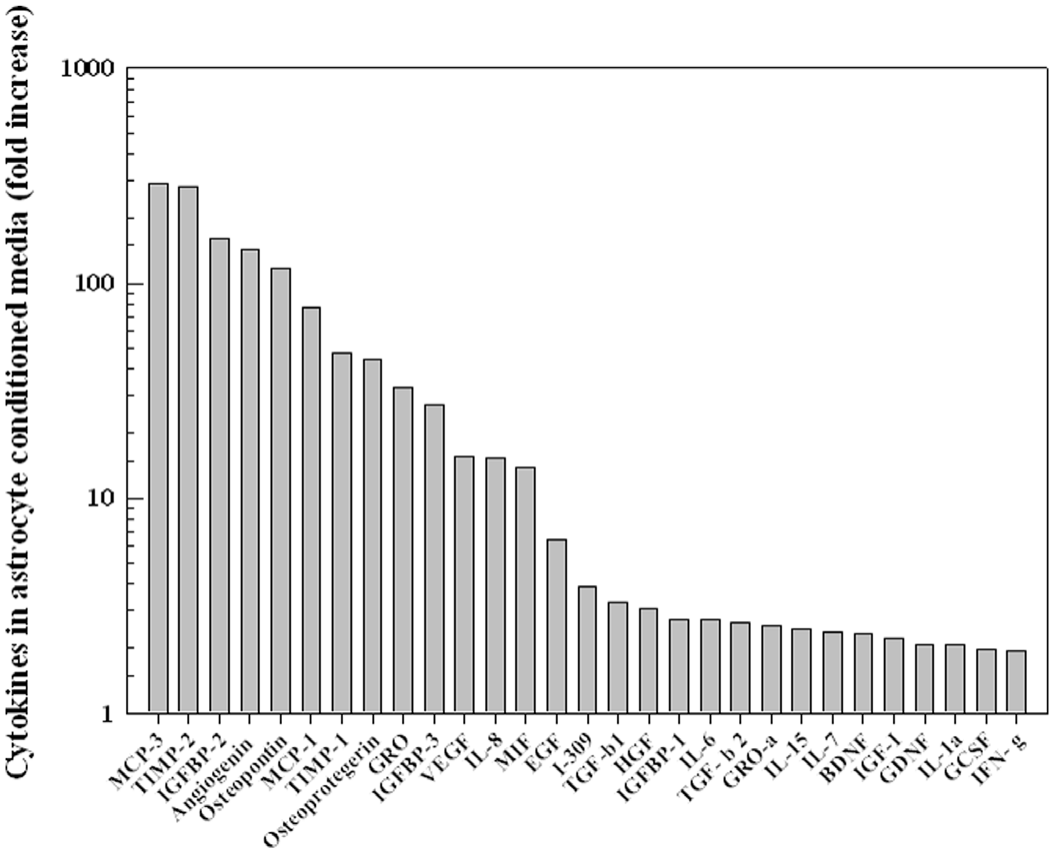
Chemokine/Cytokine profile from astrocyte conditioned media. Waterfall diagram comparing chemokines/cytokines detected in astrocyte conditioned media as compared to stem cell medium. Values shown represent the level of proteins in astrocyte conditioned media with a greater >2-fold-change compared to stem cell medium alone.

To begin to address the mechanisms through which astrocytes could influence GSC invasion capacity, chemokine/cytokine profiling was performed on astrocyte conditioned media. In this analysis stem cell medium was conditioned on astrocyte cultures for 48 h, the same procedure as used to generate the CM in Figure 1, and compared to stem cell medium alone. As shown in Figure 6 the levels of 13 bioactive molecules were increased at least 10-fold and an additional 16 increased at least 2-fold in the media conditioned on astrocytes. Of those molecules a number of have been associated with cell migration/invasion, tissue remodeling and inflammatory response, processes that are likely to influence tumor cell invasion, including MCP-3 (CCL7), osteopontin [31], TGFB-1 [19], IL-6 [32] and IL-8 [32]. These data suggest that proteins secreted by astrocytes may play a role in mediating CD133+ cell invasion.

## Discussion

GSCs are now considered to play a major role in GBM biology. Given that infiltration into normal parenchyma is a hallmark of GBMs in situ, it would seem that GSCs should also contribute to their invasive propensity. To investigate GSC invasion potential, we used two CD133+ cell lines isolated from GBM surgical specimens; as previously reported ^13–14^ these lines fit the in vitro criteria of tumor stem like cells [4]. Although additional markers of the GSC phenotype exist [33] and CD133 does not identify all GSCs, the behavior of CD133+ cells used in this study is consistent with the criteria used to GSCs in general [2], [4]. For comparison to a non-GSC phenotype, the studies described here used the differentiated progeny of the CD133+ GSCs, which provides an isogenic model system for defining the influence of the stem cell like phenotype on invasion.

Invasion is a complex process involving interactions between tumor cells with the extra-cellular matrix as well as with normal cells [34]. In an attempt to incorporate such interactions into the in vitro investigation of GBM cell invasion, we used co-cultures of normal human astrocytes with GSCs or their differentiated progeny (non-GSCs). As compared to the invasion capacity defined for each GBM cell type in isolation, the presence of astrocytes significantly increased the invasion of CD133+ GSCs yet had no effect on CD133- non GSCs. The lack of an increase in GSC invasion in co-cultures with fibroblasts suggests that there is at least some specific requirement for astrocytes. The inability of FBS, which is often used as a stimuli for tumor cell invasion [16], to enhance CD133+ cell invasion in the trans-well assay is also supportive of an astrocyte specific effect. Astrocytes serve in multiple functional roles within the CNS under normal conditions as well as in its response to a variety of injuries [9], [10]. As part of these processes astrocytes influence the behavior of neurons, brain endothelial cells and microglia [9], [10]. More recently, astrocytes have been reported to decrease the chemosensitivity of melanoma and breast tumor cell lines [35], [36]. The data presented herein suggest that an additional attribute of astrocytes is the ability to enhance GSC invasion.

Astrocytes regulate normal cells within the CNS via a number of cell-cell signaling processes [9]. The transwell experiments using astrocyte monolayers and conditioned media (Figure 1) indicate that astrocytes can enhance the invasive potential of GSCs through a paracrine based mechanism. Whereas it has long been assumed that autocrine factors expressed by GBM cells contribute to their infiltrative character [37], the data presented here suggest that secreted factors from the normal microenvironment may also contribute to GBM invasion. The indirect co-culture with astrocytes induced GSCs to express genes associated with migration and invasion such as those shown in Figures 3C and D. The number of invasion/migration associated genes whose expression was increased as a result of co-culture with astrocytes suggests that there are multiple molecules and processes acting in combination or in a redundant manner to stimulate GSC invasion under in vivo conditions.

Towards defining astrocyte provided paracrine factors that could influence such GSC gene expression and invasion capacity, cytokine/chemokine profiling was performed on astrocyte conditioned media. This analysis identified a number of proteins that have not only been associated with tumor cell invasion/migration in general but also that of GBM cells including osteopontin [38], IL-6 [39], IL-8 [40] MCP-1 [41], VEGF [42], IGFBP-2 [43], GDNF [39], HGF [44], TGFB1 [45] and TGFB2 [39]. Whereas TIMP1 [46] and TIMP2 [46], proteins generally associated with inhibiting cell invasion, were also enriched in the astrocyte conditioned medium, their role in GBM biology may not be straightforward. That is, increased TIMP1 expression has been correlated with shorter overall survival of GBM patients [47] and TIMP-2 concentrations have been reported to be greater at the tumor margin [40]. Given the number of potentially relevant proteins detected in the astrocyte conditioned media, there are likely to be multiple paracrine factors acting individually and/or in a combinatorial manner to modulate GSC invasion.

The ability of GSCs to invade into an astrocyte monolayer as shown in Figure 2, an event significantly reduced for non-GSCs, suggested that in addition to secreted factors, astrocytes may also enhance GSC invasion through direct cell contact. To provide insight into the potential role of direct contact with astrocytes as a mediator of GSC invasion, the gene expression profiles generated for GSCs grown on membranes with astrocytes in the bottom well (indirect co-culture) and GSCs grown on top of astrocyte monolayer (direct co-culture) were compared. The changes in GSC gene expression resulting from direct co-culture can be the consequence of paracrine signaling or direct cell contact, which is consistent with the significant overlap in genes whose expression was increased in GSCs under both co-culture conditions. However, there was also a subset of genes that were uniquely increased under direct co-culture conditions. Of those genes unique to direct co-culture 58 could be placed in a network with hub-genes related to invasion and migration such as *ICAM1*, *TGFB2*, *NRP1*, *AXL*, *CXCL12* and *CTGF* [28], [29], [48], [49]. These data are consistent with a recent report by Edwards et al. in which orthotopic xenografts initiated from GSCs secrete *CTGF*, a cytokine they linked to GBM invasion [50]. These results suggest that astrocytes influence the expression of a variety of invasion associated genes in GSCs and that the process is mediated by paracrine signaling as well as direct cell contact.

Assuming that the co-culture conditions used this study roughly simulate one aspect of the in situ brain environment, the data presented here are consistent with a critical role of GSCs in the infiltrative propensity of GBMs. Clearly, there are other normal CNS phenotypes (e.g. neurons, oligodendrocytes and microglia) that may also influence GSC invasion/migration and may be amenable to in vitro investigations using a co-culture approach. However, the results generated using astrocytes do suggest that to understand the biology underlying the invasive behavior of GBM and to develop therapeutic strategies for limiting such invasion it will be necessary to account for the normal tissue microenvironment.

## Supporting Information

Figure S1 Influence of astrocyte co-culture on GSC CD133 expression. GSCs (NSC11 and GBAM1) were grown alone or in indirect co-culture with astrocytes for 48 h; CD133 levels were then visualized using immunofluorescence with nuclei counter-stained with DAPI.
(TIF)

Figure S2 Interconnecting network formed by 229 genes whose expression was induced by direct co-culture of NSC11 and GBAM1 GSCs with astrocytes.
(TIF)

Figure S3 Gene expression changes induced in CD133 negative NSC11 and GBAM1 cells after co-culture with astrocytes. A) Venn diagram comparing the commonly induced genes in CD133- NCS11 and GBAM1 cells exposed to astrocytes under direct and indirect co-culture conditions. B) Genes commonly increased in CD133- NSC11 and GBAM1 cells under either co-culture condition (1033) were subjected to IPA; the top five networks are shown. C) Genes commonly increased in CD133+ NSC11 and GBAM1 cells (GSCs) under either co-culture condition (286) were subjected to IPA; the top five networks are shown.
(TIF)

Table S1 Commonly affected genes (117) in NSC11 and GBAM1 GSCs after indirect co-culture with astrocytes, with highlighted genes present in network Fig. 2C.
(DOCX)

Table S2 Genes (229) whose expression was commonly affected after direct co-culture of NSC11 and GBAM1 GSCs with astrocytes (Fig. 4A).
(DOCX)

Table S3 Genes (169) whose expression was commonly affected only after the direct co-culture of NSC11 and GBAM1 GSCs with astrocytes. Highlighted genes are present in the network shown in Fig. 5C.
(DOCX)

## References

[1] Stupp R, Hegi ME, Mason WP, van den Bent MJ, Taphoorn MJ, et al (2009) Effects of radiotherapy with concomitant and adjuvant temozolomide versus radiotherapy alone on survival in glioblastoma in a randomised phase III study: 5-year analysis of the EORTC-NCIC trial. Lancet Oncol 10: 459–466. 19269895 10.1016/S1470-2045(09)70025-7

[2] Singh SK, Hawkins C, Clarke ID, Squire JA, Bayani J, et al (2004) Identification of human brain tumour initiating cells. Nature 432: 396–401. 15549107 10.1038/nature03128

[3] Galli R, Binda E, Orfanelli U, Cipelletti B, Gritti A, et al (2004) Isolation and characterization of tumorigenic, stem-like neural precursors from human glioblastoma. Cancer Res 64: 7011–7021. 15466194 10.1158/0008-5472.CAN-04-1364

[4] Singh SK, Clarke ID, Terasaki M, Bonn VE, Hawkins C, et al (2003) Identification of a cancer stem cell in human brain tumors. Cancer Res 63: 5821–5828. 14522905

[5] Cheng L, Wu Q, Guryanova OA, Huang Z, Huang Q, et al (2011) Elevated invasive potential of glioblastoma stem cells. Biochem Biophys Res Commun 406: 643–648. 21371437 10.1016/j.bbrc.2011.02.123 PMC3065536

[6] Tower DB, Young OM (1973) The activities of butyrylcholinesterase and carbonic anhydrase, the rate of anaerobic glycolysis, and the question of a constant density of glial cells in cerebral cortices of various mammalian species from mouse to whale. J Neurochem 20: 269–278. 4633361 10.1111/j.1471-4159.1973.tb12126.x

[7] Garcia-Segura LM, McCarthy MM (2004) Minireview: Role of glia in neuroendocrine function. Endocrinology 145: 1082–1086. 14670989 10.1210/en.2003-1383

[8] Aloisi F, Borsellino G, Care A, Testa U, Gallo P, et al (1995) Cytokine regulation of astrocyte function: in-vitro studies using cells from the human brain. Int J Dev Neurosci 13: 265–274. 7572280 10.1016/0736-5748(94)00071-a

[9] Sofroniew MV, Vinters HV (2010) Astrocytes: biology and pathology. Acta Neuropathol 119: 7–35. 20012068 10.1007/s00401-009-0619-8 PMC2799634

[10] Chen Y, Swanson RA (2003) Astrocytes and brain injury. J Cereb Blood Flow Metab 23: 137–149. 12571445 10.1097/01.WCB.0000044631.80210.3C

[11] Ethell IM, Ethell DW (2007) Matrix metalloproteinases in brain development and remodeling: synaptic functions and targets. J Neurosci Res 85: 2813–2823. 17387691 10.1002/jnr.21273

[12] Bellail AC, Hunter SB, Brat DJ, Tan C, Van Meir EG (2004) Microregional extracellular matrix heterogeneity in brain modulates glioma cell invasion. Int J Biochem Cell Biol 36: 1046–1069. 15094120 10.1016/j.biocel.2004.01.013

[13] McCord AM, Jamal M, Shankavaram UT, Lang FF, Camphausen K, et al (2009) Physiologic oxygen concentration enhances the stem-like properties of CD133+ human glioblastoma cells in vitro. Mol Cancer Res 7: 489–497. 19372578 10.1158/1541-7786.MCR-08-0360 PMC6290460

[14] McCord AM, Jamal M, Williams ES, Camphausen K, Tofilon PJ (2009) CD133+ glioblastoma stem-like cells are radiosensitive with a defective DNA damage response compared with established cell lines. Clin Cancer Res 15: 5145–5153. 19671863 10.1158/1078-0432.CCR-09-0263 PMC6290462

[15] Pollard SM, Yoshikawa K, Clarke ID, Danovi D, Stricker S, et al (2009) Glioma stem cell lines expanded in adherent culture have tumor-specific phenotypes and are suitable for chemical and genetic screens. Cell Stem Cell 4: 568–580. 19497285 10.1016/j.stem.2009.03.014

[16] Shaw LM (2005) Tumor cell invasion assays. Methods Mol Biol 294: 97–105. 15576908 10.1385/1-59259-860-9:097

[17] Soria G, Ofri-Shahak M, Haas I, Yaal-Hahoshen N, Leider-Trejo L, et al (2011) Inflammatory mediators in breast cancer: coordinated expression of TNFalpha & IL-1beta with CCL2 & CCL5 and effects on epithelial-to-mesenchymal transition. BMC Cancer 11: 130. 21486440 10.1186/1471-2407-11-130 PMC3095565

[18] Chetty C, Vanamala SK, Gondi CS, Dinh DH, Gujrati M, et al (2012) MMP-9 induces CD44 cleavage and CD44 mediated cell migration in glioblastoma xenograft cells. Cell Signal 24: 549–559. 22024282 10.1016/j.cellsig.2011.10.008 PMC3481542

[19] de Graauw M, van Miltenburg MH, Schmidt MK, Pont C, Lalai R, et al (2010) Annexin A1 regulates TGF-beta signaling and promotes metastasis formation of basal-like breast cancer cells. Proc Natl Acad Sci U S A 107: 6340–6345. 20308542 10.1073/pnas.0913360107 PMC2852023

[20] Tatenhorst L, Rescher U, Gerke V, Paulus W (2006) Knockdown of annexin 2 decreases migration of human glioma cells in vitro. Neuropathol Appl Neurobiol 32: 271–277. 16640645 10.1111/j.1365-2990.2006.00720.x

[21] Udabage L, Brownlee GR, Waltham M, Blick T, Walker EC, et al (2005) Antisense-mediated suppression of hyaluronan synthase 2 inhibits the tumorigenesis and progression of breast cancer. Cancer Res 65: 6139–6150. 16024615 10.1158/0008-5472.CAN-04-1622

[22] Sen S, Dong M, Kumar S (2009) Isoform-specific contributions of alpha-actinin to glioma cell mechanobiology. PLoS One 4: e8427. 20037648 10.1371/journal.pone.0008427 PMC2793025

[23] Rodrigues-Ferreira S, Abdelkarim M, Dillenburg-Pilla P, Luissint AC, di-Tommaso A, et al (2012) Angiotensin II facilitates breast cancer cell migration and metastasis. PLoS One 7: e35667. 22536420 10.1371/journal.pone.0035667 PMC3334979

[24] Udabage L, Brownlee GR, Nilsson SK, Brown TJ (2005) The over-expression of HAS2, Hyal-2 and CD44 is implicated in the invasiveness of breast cancer. Exp Cell Res 310: 205–217. 16125700 10.1016/j.yexcr.2005.07.026

[25] Kohutek ZA, diPierro CG, Redpath GT, Hussaini IM (2009) ADAM-10-mediated N-cadherin cleavage is protein kinase C-alpha dependent and promotes glioblastoma cell migration. J Neurosci 29: 4605–4615. 19357285 10.1523/JNEUROSCI.5126-08.2009 PMC3133728

[26] Liu Q, Li G, Li R, Shen J, He Q, et al (2010) IL-6 promotion of glioblastoma cell invasion and angiogenesis in U251 and T98G cell lines. J Neurooncol 100: 165–176. 20361349 10.1007/s11060-010-0158-0

[27] Slack-Davis JK, Atkins KA, Harrer C, Hershey ED, Conaway M (2009) Vascular cell adhesion molecule-1 is a regulator of ovarian cancer peritoneal metastasis. Cancer Res 69: 1469–1476. 19208843 10.1158/0008-5472.CAN-08-2678

[28] Demuth T, Rennert JL, Hoelzinger DB, Reavie LB, Nakada M, et al (2008) Glioma cells on the run - the migratory transcriptome of 10 human glioma cell lines. BMC Genomics 9: 54. 18230158 10.1186/1471-2164-9-54 PMC2275271

[29] Wick W, Grimmel C, Wild-Bode C, Platten M, Arpin M, et al (2001) Ezrin-dependent promotion of glioma cell clonogenicity, motility, and invasion mediated by BCL-2 and transforming growth factor-beta2. J Neurosci 21: 3360–3368. 11331365 10.1523/JNEUROSCI.21-10-03360.2001 PMC6762489

[30] Ehtesham M, Winston JA, Kabos P, Thompson RC (2006) CXCR4 expression mediates glioma cell invasiveness. Oncogene 25: 2801–2806. 16407848 10.1038/sj.onc.1209302

[31] Wang D, Yamamoto S, Hijiya N, Benveniste EN, Gladson CL (2000) Transcriptional regulation of the human osteopontin promoter: functional analysis and DNA-protein interactions. Oncogene 19: 5801–5809. 11126367 10.1038/sj.onc.1203917

[32] Seike T, Fujita K, Yamakawa Y, Kido MA, Takiguchi S, et al (2011) Interaction between lung cancer cells and astrocytes via specific inflammatory cytokines in the microenvironment of brain metastasis. Clin Exp Metastasis 28: 13–25. 20953899 10.1007/s10585-010-9354-8 PMC2998640

[33] Son MJ, Woolard K, Nam DH, Lee J, Fine HA (2009) SSEA-1 is an enrichment marker for tumor-initiating cells in human glioblastoma. Cell Stem Cell 4: 440–452. 19427293 10.1016/j.stem.2009.03.003 PMC7227614

[34] Friedl P, Alexander S (2011) Cancer invasion and the microenvironment: plasticity and reciprocity. Cell 147: 992–1009. 22118458 10.1016/j.cell.2011.11.016

[35] Kim SJ, Kim JS, Park ES, Lee JS, Lin Q, et al (2011) Astrocytes upregulate survival genes in tumor cells and induce protection from chemotherapy. Neoplasia 13: 286–298. 21390191 10.1593/neo.11112 PMC3050871

[36] Lin Q, Balasubramanian K, Fan D, Kim SJ, Guo L, et al (2010) Reactive astrocytes protect melanoma cells from chemotherapy by sequestering intracellular calcium through gap junction communication channels. Neoplasia 12: 748–754. 20824051 10.1593/neo.10602 PMC2933695

[37] Hoelzinger DB, Demuth T, Berens ME (2007) Autocrine factors that sustain glioma invasion and paracrine biology in the brain microenvironment. J Natl Cancer Inst 99: 1583–1593. 17971532 10.1093/jnci/djm187

[38] Jan HJ, Lee CC, Shih YL, Hueng DY, Ma HI, et al (2010) Osteopontin regulates human glioma cell invasiveness and tumor growth in mice. Neuro Oncol 12: 58–70. 20150368 10.1093/neuonc/nop013 PMC2940564

[39] Jones TS, Holland EC (2011) Standard of care therapy for malignant glioma and its effect on tumor and stromal cells. Oncogene. 10.1038/onc.2011.398 21909136

[40] Marcus HJ, Carpenter KL, Price SJ, Hutchinson PJ (2010) In vivo assessment of high-grade glioma biochemistry using microdialysis: a study of energy-related molecules, growth factors and cytokines. J Neurooncol 97: 11–23. 19714445 10.1007/s11060-009-9990-5

[41] Liang Y, Bollen AW, Gupta N (2008) CC chemokine receptor-2A is frequently overexpressed in glioblastoma. J Neurooncol 86: 153–163. 17703277 10.1007/s11060-007-9463-7

[42] Hong X, Jiang F, Kalkanis SN, Zhang ZG, Zhang XP, et al (2006) SDF-1 and CXCR4 are up-regulated by VEGF and contribute to glioma cell invasion. Cancer Lett 236: 39–45. 15967572 10.1016/j.canlet.2005.05.011

[43] Song SW, Fuller GN, Khan A, Kong S, Shen W, et al (2003) IIp45, an insulin-like growth factor binding protein 2 (IGFBP-2) binding protein, antagonizes IGFBP-2 stimulation of glioma cell invasion. Proc Natl Acad Sci U S A 100: 13970–13975. 14617774 10.1073/pnas.2332186100 PMC283530

[44] Esencay M, Newcomb EW, Zagzag D (2010) HGF upregulates CXCR4 expression in gliomas via NF-kappaB: implications for glioma cell migration. J Neurooncol 99: 33–40. 20157762 10.1007/s11060-010-0111-2 PMC3767998

[45] Anido J, Saez-Borderias A, Gonzalez-Junca A, Rodon L, Folch G, et al (2010) TGF-beta Receptor Inhibitors Target the CD44(high)/Id1(high) Glioma-Initiating Cell Population in Human Glioblastoma. Cancer Cell 18: 655–668. 21156287 10.1016/j.ccr.2010.10.023

[46] Mohanam S, Wang SW, Rayford A, Yamamoto M, Sawaya R, et al (1995) Expression of tissue inhibitors of metalloproteinases: negative regulators of human glioblastoma invasion in vivo. Clin Exp Metastasis 13: 57–62. 7820957 10.1007/BF00144019

[47] Aaberg-Jessen C, Christensen K, Offenberg H, Bartels A, Dreehsen T, et al (2009) Low expression of tissue inhibitor of metalloproteinases-1 (TIMP-1) in glioblastoma predicts longer patient survival. J Neurooncol 95: 117–128. 19430729 10.1007/s11060-009-9910-8

[48] Vajkoczy P, Knyazev P, Kunkel A, Capelle HH, Behrndt S, et al (2006) Dominant-negative inhibition of the Axl receptor tyrosine kinase suppresses brain tumor cell growth and invasion and prolongs survival. Proc Natl Acad Sci U S A 103: 5799–5804. 16585512 10.1073/pnas.0510923103 PMC1458653

[49] Folkins C, Shaked Y, Man S, Tang T, Lee CR, et al (2009) Glioma tumor stem-like cells promote tumor angiogenesis and vasculogenesis via vascular endothelial growth factor and stromal-derived factor 1. Cancer Res 69: 7243–7251. 19738068 10.1158/0008-5472.CAN-09-0167 PMC3409689

[50] Edwards LA, Woolard K, Son MJ, Li A, Lee J, et al (2011) Effect of brain- and tumor-derived connective tissue growth factor on glioma invasion. J Natl Cancer Inst 103: 1162–1178. 21771732 10.1093/jnci/djr224 PMC3149042

