# Supplementary figures and images for: Astrocytes Enhance the Invasion Potential of Glioblastoma Stem-Like Cells
Source: PLoS One. 2013 Jan 22;8(1):e54752. doi: 10.1371/journal.pone.0054752 (PMC3551925; doi:10.1371/journal.pone.0054752)

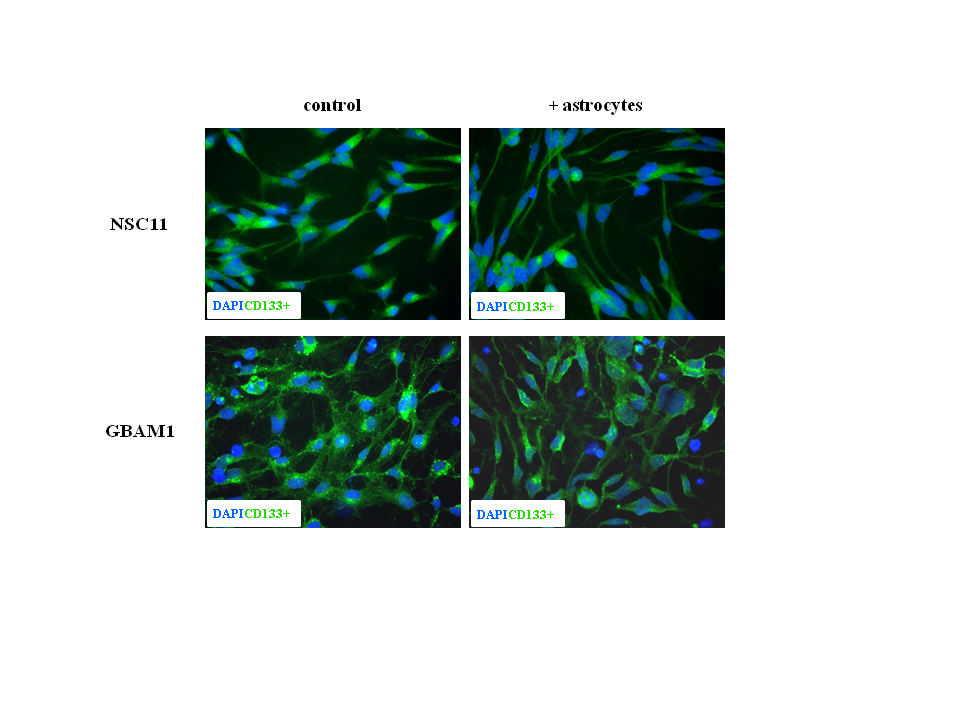

Supplement: Figure S1 — Influence of astrocyte co-culture on GSC CD133 expression. GSCs (NSC11 and GBAM1) were grown alone or in indirect co-culture with astrocytes for 48 h; CD133 levels were then visualized using immunofluorescence with nuclei counter-stained with DAPI. (TIF) [file pone.0054752.s001.tif]

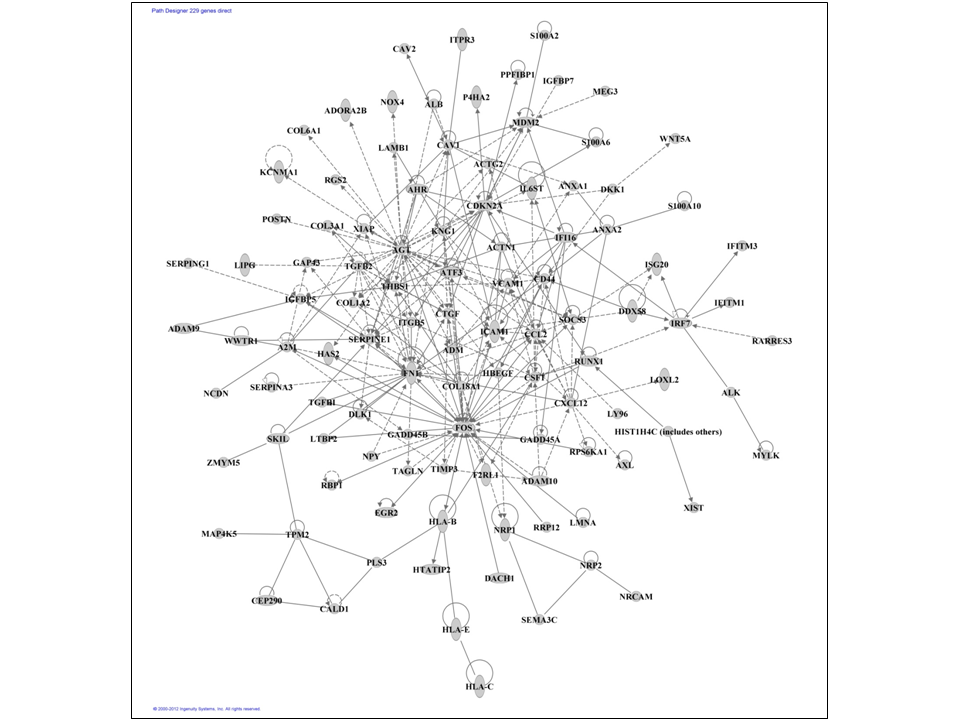

Supplement: Figure S2 — Interconnecting network formed by 229 genes whose expression was induced by direct co-culture of NSC11 and GBAM1 GSCs with astrocytes. (TIF) [file pone.0054752.s002.tif]
