# Supplementary material for: Astrocytes Enhance the Invasion Potential of Glioblastoma Stem-Like Cells
Source: PLoS One. 2013 Jan 22;8(1):e54752. doi: 10.1371/journal.pone.0054752 (PMC3551925; doi:10.1371/journal.pone.0054752)
Supplement: Table S1 — Commonly affected genes (117) in NSC11 and GBAM1 GSCs after indirect co-culture with astrocytes, with highlighted genes present in network Fig. 2C. (DOCX) [file pone.0054752.s004.docx]

Table S1. Commonly affected genes (117) in NSC11 and GBAM1 GSCs after indirect co-culture with astrocytes, with highlighted genes present in network Fig. 2C.

| **A2M** | **alpha-2-macroglobulin** | |  |  |
| --- | --- | --- | --- | --- |
| **ACTG2** | **actin, gamma 2, smooth muscle, enteric** | |  |  |
| **ACTN1** | **actinin, alpha 1** | |  |  |
| **ADM** | **adrenomedullin** | |  |  |
| **AGT** | **angiotensinogen (serpin peptidase inhibitor, clade A, member 8)** | |  |  |
| ANX2P2 | annexin A2 pseudogene 2 | |  |  |
| **ANXA1** | **annexin A1** | |  |  |
| **ANXA2** | **annexin A2** | |  |  |
| ANXA2P3 | annexin A2 pseudogene 3 | |  |  |
| **AP1S2** | **adaptor-related protein complex 1, sigma 2 subunit** | |  |  |
| APOBEC3B | apolipoprotein B mRNA editing enzyme, catalytic polypeptide-like 3B | |  |  |
| ARL4C | ADP-ribosylation factor-like 4C | |  |  |
| ARPC1B | actin related protein 2/3 complex, subunit 1B, 41kDa | |  |  |
| **ATF3** | **activating transcription factor 3** | |  |  |
| AZI1 | 5-azacytidine induced 1 | |  |  |
| BACE2 | beta-site APP-cleaving enzyme 2 | |  |  |
| BAG3 | BCL2-associated athanogene 3 | |  |  |
| **BATF3** | **basic leucine zipper transcription factor, ATF-like 3** | |  |  |
| BEAN1 | brain expressed, associated with NEDD4, 1 | |  |  |
| CADM4 | cell adhesion molecule 4 | |  |  |
| **CCL2** | **chemokine (C-C motif) ligand 2** | |  |  |
| **CD44** | **CD44 molecule (Indian blood group)** | |  |  |
| CHRNG | cholinergic receptor, nicotinic, gamma | |  |  |
| CHST2 | carbohydrate (N-acetylglucosamine-6-O) sulfotransferase 2 | |  |  |
| **CSF1** | **colony stimulating factor 1 (macrophage)** | |  |  |
| **DDIT3** | **DNA-damage-inducible transcript 3** | |  |  |
| **DLK1** | **delta-like 1 homolog (Drosophila)** | |  |  |
| DLX2 | distal-less homeobox 2 | |  |  |
| **DUSP5** | **dual specificity phosphatase 5** | |  |  |
| ECM1 | extracellular matrix protein 1 | |  |  |
| EMP1 | epithelial membrane protein 1 | |  |  |
| EPB41L4B | erythrocyte membrane protein band 4.1 like 4B | |  |  |
| FMO4 | flavin containing monooxygenase 4 | |  |  |
| **FOSL1** | **FOS-like antigen 1** | |  |  |
| **GADD45B** | **growth arrest and DNA-damage-inducible, beta** | |  |  |
| **GAP43** | **growth associated protein 43** | |  |  |
| **GATA4** | **GATA binding protein 4** | |  |  |
| GDAP2 | ganglioside induced differentiation associated protein 2 | |  |  |
| GLTSCR1 | glioma tumor suppressor candidate region gene 1 | |  |  |
| GRWD1 | glutamate-rich WD repeat containing 1 | |  |  |
| **HAS2** | **hyaluronan synthase 2** | |  |  |
| **HBEGF** | **heparin-binding EGF-like growth factor** | |  |  |
| HLA-C | major histocompatibility complex, class I, C | |  |  |
| HLA-DRB4 | major histocompatibility complex, class II, DR beta 4 | |  |  |
| **HMGA1** | **high mobility group AT-hook 1** | |  |  |
| HPCAL4 | hippocalcin like 4 | |  |  |
| HS3ST3A1 | heparan sulfate (glucosamine) 3-O-sulfotransferase 3A1 | |  |  |
| HTATIP2 | HIV-1 Tat interactive protein 2, 30kDa | |  |  |
| IDS | iduronate 2-sulfatase | |  |  |
| **IFI16** | **interferon, gamma-inducible protein 16** | |  |  |
| **IFITM2** | **interferon induced transmembrane protein 2 (1-8D)** | |  |  |
| **IGFBP5** | **insulin-like growth factor binding protein 5** | |  |  |
| IL13RA1 | interleukin 13 receptor, alpha 1 | |  |  |
| **IRF7** | **interferon regulatory factor 7** | |  |  |
| ITK | IL2-inducible T-cell kinase | |  |  |
| JMJD4 | jumonji domain containing 4 | |  |  |
| KCNF1 | potassium voltage-gated channel, subfamily F, member 1 | |  |  |
| KIAA0894 | KIAA0894 protein | |  |  |
| **KRT8** | **keratin 8** |  |  |  |
| LMNA | lamin A/C |  |  |  |
| LOC729659 | S100 calcium-binding protein A14 (calgizzarin) |  |  |  |
| LOC92249 | hypothetical LOC92249 |  |  |  |
| LSR | lipolysis stimulated lipoprotein receptor |  |  |  |
| **LY96** | **lymphocyte antigen 96** |  |  |  |
| LZTS1 | leucine zipper, putative tumor suppressor 1 |  |  |  |
| MED13L | mediator complex subunit 13-like |  |  |  |
| MGC4771 | hypothetical protein MGC4771 |  |  |  |
| **NCDN** | **neurochondrin** |  |  |  |
| NLE1 | notchless homolog 1 (Drosophila) |  |  |  |
| NNMT | nicotinamide N-methyltransferase |  |  |  |
| **NOV** | **nephroblastoma overexpressed gene** |  |  |  |
| NPTX2 | neuronal pentraxin II |  |  |  |
| NPY | neuropeptide Y |  |  |  |
| PCYT1B | phosphate cytidylyltransferase 1, choline, beta |  |  |  |
| PDLIM4 | PDZ and LIM domain 4 |  |  |  |
| PHLDA2 | pleckstrin homology-like domain, family A, member 2 |  |  |  |
| PLEKHA4 | pleckstrin homology domain containing, family A (phosphoinositide binding specific) member 4 |  |  |  |
| PLP2 | proteolipid protein 2 (colonic epithelium-enriched) |  |  |  |
| PRSS23 | protease, serine, 23 |  |  |  |
| **PSMB8** | **proteasome (prosome, macropain) subunit, beta type, 8 (large multifunctional peptidase 7)** |  |  |  |
| **PSMB9** | **proteasome (prosome, macropain) subunit, beta type, 9 (large multifunctional peptidase 2)** |  |  |  |
| **PTGES** | **prostaglandin E synthase** |  |  |  |
| **PTGS1** | **prostaglandin-endoperoxide synthase 1 (prostaglandin G/H synthase and cyclooxygenase)** |  |  |  |
| PTRF | polymerase I and transcript release factor |  |  |  |
| PURG | purine-rich element binding protein G |  |  |  |
| RASL11B | RAS-like, family 11, member B |  |  |  |
| RGS10 | regulator of G-protein signaling 10 |  |  |  |
| RNASEH2B | ribonuclease H2, subunit B |  |  |  |
| RPL10L | ribosomal protein L10-like |  |  |  |
| **S100A10** | **S100 calcium binding protein A10** |  |  |  |
| **S100A11** | **S100 calcium binding protein A11** |  |  |  |
| S100A2 | S100 calcium binding protein A2 |  |  |  |
| S100A6 | S100 calcium binding protein A6 |  |  |  |
| SCG2 | secretogranin II |  |  |  |
| SERPINA3 | serpin peptidase inhibitor, clade A (alpha-1 antiproteinase, antitrypsin), member 3 |  |  |  |
| **SERPINE1** | **serpin peptidase inhibitor, clade E (nexin, plasminogen activator inhibitor type 1), member 1** |  |  |  |
| **SERPING1** | **serpin peptidase inhibitor, clade G (C1 inhibitor), member 1** |  |  |  |
| SGK1 | serum/glucocorticoid regulated kinase 1 |  |  |  |
| SH3BP5 | SH3-domain binding protein 5 (BTK-associated) |  |  |  |
| **SOCS3** | **suppressor of cytokine signaling 3** |  |  |  |
| SOCS6 | suppressor of cytokine signaling 6 |  |  |  |
| SRPX | sushi-repeat containing protein, X-linked |  |  |  |
| SVIL | supervillin |  |  |  |
| TAF13 | TAF13 RNA polymerase II, TATA box binding protein (TBP)-associated factor, 18kDa |  |  |  |
| TAGLN2 | transgelin 2 |  |  |  |
| **THBS1** | **thrombospondin 1** |  |  |  |
| TMEM110 | transmembrane protein 110 |  |  |  |
| TMEM158 | transmembrane protein 158 (gene/pseudogene) |  |  |  |
| **TNFRSF12A** | **tumor necrosis factor receptor superfamily, member 12A** |  |  |  |
| TPK1 | thiamin pyrophosphokinase 1 |  |  |  |
| TRA@ | T cell receptor alpha locus |  |  |  |
| TRAF3IP2 | TRAF3 interacting protein 2 |  |  |  |
| VWA5A | von Willebrand factor A domain containing 5A |  |  |  |
| WDR4 | WD repeat domain 4 |  |  |  |
| WDR61 | WD repeat domain 61 |  |  |  |
| ZNF250 | zinc finger protein 250 |  |  |  |
| ZNF365 | zinc finger protein 365 |  |  |  |
