# Supplementary material for: Astrocytes Enhance the Invasion Potential of Glioblastoma Stem-Like Cells
Source: PLoS One. 2013 Jan 22;8(1):e54752. doi: 10.1371/journal.pone.0054752 (PMC3551925; doi:10.1371/journal.pone.0054752)
Supplement: Table S2 — Genes (229) whose expression was commonly affected after direct co-culture of NSC11 and GBAM1 GSCs with astrocytes (Fig. 4A). (DOCX) [file pone.0054752.s005.docx]

Table S2. Genes (229) whose expression was commonly affected after direct co-culture of NSC11 and GBAM1 GSCs with astrocytes (Fig. 4A).

| A2M | alpha-2-macroglobulin |
| --- | --- |
| ACTG2 | actin, gamma 2, smooth muscle, enteric |
| ACTN1 | actinin, alpha 1 |
| ACTN2 | actinin, alpha 2 |
| ADAM10 | a disintegrin and metalloproteinase domain 10 |
| ADAM9 | ADAM metallopeptidase domain 9 (meltrin gamma) |
| ADFP | adipose differentiation-related protein |
| ADM | adrenomedullin |
| ADORA2B | adenosine A2b receptor |
| AGT | angiotensinogen (serpin peptidase inhibitor, clade A, member 8) |
| AHR | aryl hydrocarbon receptor |
| AIM1 | absent in melanoma 1 |
| ALB | albumin |
| ALK | anaplastic lymphoma kinase (Ki-1) |
| AMIGO2 | adhesion molecule with Ig-like domain 2 |
| ANKRD26 | ankyrin repeat domain 26 |
| ANXA1 | annexin A1 |
| ANXA2 | annexin A2 |
| ANXA2P2 | annexin A2 pseudogene 2 |
| ARID5A | AT rich interactive domain 5A (MRF1-like) |
| ARL4C | ADP-ribosylation factor-like 4C |
| ATF3 | activating transcription factor 3 |
| ATHL1 | ATH1, acid trehalase-like 1 (yeast) |
| AXL | AXL receptor tyrosine kinase |
| BACE2 | beta-site APP-cleaving enzyme 2 |
| BIRC4 | baculoviral IAP repeat-containing 4 |
| BLVRB | biliverdin reductase B (flavin reductase (NADPH)) |
| C14orf78 | chromosome 14 open reading frame 78 |
| CALB1 | calbindin 1, 28kDa |
| CALD1 | caldesmon 1 |
| CAV1 | caveolin 1, caveolae protein, 22kDa |
| CAV2 | caveolin 2 |
| CCL2 | chemokine (C-C motif) ligand 2 |
| CD44 | CD44 antigen (homing function and Indian blood group system) |
| CDKN2A | cyclin-dependent kinase inhibitor 2A (melanoma, p16, inhibits CDK4) |
| CLIC2 | chloride intracellular channel 2 |
| COL13A1 | collagen, type XIII, alpha 1 |
| COL18A1 | collagen, type XVIII, alpha 1 |
| COL1A2 | collagen, type I, alpha 2 |
| COL3A1 | collagen, type III, alpha 1 (Ehlers-Danlos syndrome type IV, autosomal dominant) |
| COL6A1 | collagen, type VI, alpha 1 |
| CSF1 | colony stimulating factor 1 (macrophage) |
| CTGF | connective tissue growth factor |
| CXCL12 | chemokine (C-X-C motif) ligand 12 (stromal cell-derived factor 1) |
| DACH1 | dachshund homolog 1 (Drosophila) |
| DCAMKL2 | Doublecortin and CaM kinase-like 2 |
| DCBLD2 | discoidin, CUB and LCCL domain containing 2 |
| DDX58 | DEAD (Asp-Glu-Ala-Asp) box polypeptide 58 |
| DENND4A | DENN/MADD domain containing 4A |
| DIRAS3 | DIRAS family, GTP-binding RAS-like 3 |
| DKK1 | dickkopf homolog 1 (Xenopus laevis) |
| DLG1 | discs, large homolog 1 (Drosophila) |
| DLK1 | delta-like 1 homolog (Drosophila) |
| DNASE1L1 | deoxyribonuclease I-like 1 |
| DYM | dymeclin |
| ECM1 | extracellular matrix protein 1 |
| EEA1 | early endosome antigen 1, 162kD |
| EGR2 | early growth response 2 (Krox-20 homolog, Drosophila) |
| EMP1 | epithelial membrane protein 1 |
| F2RL1 | coagulation factor II (thrombin) receptor-like 1 |
| FABP5 | fatty acid binding protein 5 (psoriasis-associated) |
| FAM134B | family with sequence similarity 134, member B |
| FAM63B | family with sequence similarity 63, member B |
| FBN2 | fibrillin 2 (congenital contractural arachnodactyly) |
| FER1L3 | fer-1-like 3, myoferlin (C. elegans) |
| FLJ13615 | hypothetical protein FLJ13615 |
| FLJ21924 | hypothetical protein FLJ21924 |
| FLNC | filamin C, gamma (actin binding protein 280) |
| FN1 | fibronectin 1 |
| FOS | v-fos FBJ murine osteosarcoma viral oncogene homolog |
| GADD45A | growth arrest and DNA-damage-inducible, alpha |
| GADD45B | growth arrest and DNA-damage-inducible, beta |
| GAP43 | growth associated protein 43 |
| GDAP2 | ganglioside induced differentiation associated protein 2 |
| GLIPR1 | GLI pathogenesis-related 1 (glioma) |
| GLTSCR1 | glioma tumor suppressor candidate region gene 1 |
| GOLGA8B | golgi autoantigen, golgin subfamily a, 8B |
| GPNMB | glycoprotein (transmembrane) nmb |
| GPR51 | G protein-coupled receptor 51 |
| HAS2 | hyaluronan synthase 2 |
| HBEGF | heparin-binding EGF-like growth factor |
| HERC4 | hect domain and RLD 4 |
| HIST1H4E | histone 1, H4e |
| HLA-B | major histocompatibility complex, class I, B |
| HLA-C | major histocompatibility complex, class I, C |
| HLA-E | major histocompatibility complex, class I, E |
| HRASLS3 | HRAS-like suppressor 3 |
| HS3ST3A1 | heparan sulfate (glucosamine) 3-O-sulfotransferase 3A1 |
| HSPA2 | heat shock 70kDa protein 2 |
| HTATIP2 | HIV-1 Tat interactive protein 2, 30kDa |
| ICAM1 | intercellular adhesion molecule 1 (CD54), human rhinovirus receptor |
| IER3 | immediate early response 3 |
| IF | I factor (complement) |
| IFI16 | interferon, gamma-inducible protein 16 |
| IFITM1 | interferon induced transmembrane protein 1 (9-27) |
| IFITM3 | interferon induced transmembrane protein 3 (1-8U) |
| IGFBP5 | insulin-like growth factor binding protein 5 |
| IGFBP7 | insulin-like growth factor binding protein 7 |
| IL13RA1 | interleukin 13 receptor, alpha 1 |
| IL6ST | interleukin 6 signal transducer (gp130, oncostatin M receptor) |
| IRF7 | interferon regulatory factor 7 |
| ISG20 | interferon stimulated exonuclease gene 20kDa |
| ITGB5 | integrin, beta 5 |
| ITPR3 | inositol 1,4,5-triphosphate receptor, type 3 |
| KCNF1 | potassium voltage-gated channel, subfamily F, member 1 |
| KCNMA1 | potassium large conductance calcium-activated channel, subfamily M, alpha member 1 |
| KIAA0367 | KIAA0367 |
| KIAA0690 | KIAA0690 |
| KIAA0830 | KIAA0830 protein |
| KIAA1033 | KIAA1033 |
| KIAA1164 | hypothetical protein KIAA1164 |
| KNG | Kininogen |
| LAMB1 | laminin, beta 1 |
| LDLRAP1 | low density lipoprotein receptor adaptor protein 1 |
| LEPREL2 | leprecan-like 2 |
| LIPG | lipase, endothelial |
| LMNA | lamin A/C |
| LOC283687 | hypothetical protein LOC283687 |
| LOC54103 | hypothetical protein LOC54103 |
| LOC92249 | hypothetical protein LOC92249 |
| LOH11CR2A | loss of heterozygosity, 11, chromosomal region 2, gene A |
| LOXL1 | lysyl oxidase-like 1 |
| LOXL2 | lysyl oxidase-like 2 |
| LTBP2 | latent transforming growth factor beta binding protein 2 |
| LY96 | lymphocyte antigen 96 |
| LZTS1 | leucine zipper, putative tumor suppressor 1 |
| MALT1 | mucosa associated lymphoid tissue lymphoma translocation gene 1 |
| MAN2A1 | mannosidase, alpha, class 2A, member 1 |
| MAOB | monoamine oxidase B |
| MAP3K6 | mitogen-activated protein kinase kinase kinase 6 |
| MAP4K5 | mitogen-activated protein kinase kinase kinase kinase 5 |
| MDFIC | MyoD family inhibitor domain containing |
| MDM2 | Mdm2, transformed 3T3 cell double minute 2, p53 binding protein (mouse) |
| ME1 | malic enzyme 1, NADP(+)-dependent, cytosolic |
| MEG3 | maternally expressed 3 |
| MFAP5 | microfibrillar associated protein 5 |
| MGC12262 | hypothetical protein MGC12262 |
| MICALL2 | MICAL-like 2 |
| MLPH | melanophilin |
| MVP | major vault protein |
| MXRA5 | matrix-remodelling associated 5 |
| MXRA8 | matrix-remodelling associated 8 |
| MYLK | myosin, light polypeptide kinase |
| MYO1B | myosin IB |
| NCDN | neurochondrin |
| NDN | necdin homolog (mouse) |
| NME5 | non-metastatic cells 5, protein expressed in (nucleoside-diphosphate kinase) |
| NNMT | nicotinamide N-methyltransferase |
| NOX4 | NADPH oxidase 4 |
| NPY | neuropeptide Y |
| NRCAM | neuronal cell adhesion molecule |
| NRP1 | neuropilin 1 |
| NRP2 | neuropilin 2 |
| NT5DC3 | 5'-nucleotidase domain containing 3 |
| NT5E | 5'-nucleotidase, ecto (CD73) |
| ODZ4 | odz, odd Oz/ten-m homolog 4 (Drosophila) |
| OGFRL1 | opioid growth factor receptor-like 1 |
| P4HA2 | procollagen-proline, 2-oxoglutarate 4-dioxygenase (proline 4-hydroxylase), alpha polypeptide II |
| PAR5 | Prader-Willi/Angelman syndrome-5 |
| PHF11 | PHD finger protein 11 |
| PHLDA2 | pleckstrin homology-like domain, family A, member 2 |
| PLEKHA1 | pleckstrin homology domain containing, family A (phosphoinositide binding specific) member 1 |
| PLEKHA4 | pleckstrin homology domain containing, family A (phosphoinositide binding specific) member 4 |
| PLEKHA5 | pleckstrin homology domain containing, family A member 5 |
| PLS3 | plastin 3 (T isoform) |
| POLD4 | polymerase (DNA-directed), delta 4 |
| POSTN | periostin, osteoblast specific factor |
| PPFIBP1 | PTPRF interacting protein, binding protein 1 (liprin beta 1) |
| PPM2C | protein phosphatase 2C, magnesium-dependent, catalytic subunit |
| PPP4R2 | protein phosphatase 4, regulatory subunit 2 |
| PRSS23 | protease, serine, 23 |
| PURG | purine-rich element binding protein G |
| RAB20 | RAB20, member RAS oncogene family |
| RARRES3 | retinoic acid receptor responder (tazarotene induced) 3 |
| RBP1 | retinol binding protein 1, cellular |
| RFX3 | regulatory factor X, 3 (influences HLA class II expression) |
| RGS17 | regulator of G-protein signalling 17 |
| RGS2 | regulator of G-protein signalling 2, 24kDa |
| RPS6KA1 | ribosomal protein S6 kinase, 90kDa, polypeptide 1 |
| RUNX1 | runt-related transcription factor 1 (acute myeloid leukemia 1; aml1 oncogene) |
| S100A10 | S100 calcium binding protein A10 (annexin II ligand, calpactin I, light polypeptide (p11)) |
| S100A2 | S100 calcium binding protein A2 |
| S100A6 | S100 calcium binding protein A6 (calcyclin) |
| SCG2 | secretogranin II (chromogranin C) |
| SEC24A | SEC24 related gene family, member A (S. cerevisiae) |
| SEMA3C | sema domain, immunoglobulin domain (Ig), short basic domain, secreted, (semaphorin) 3C |
| SERPINA3 | serpin peptidase inhibitor, clade A (alpha-1 antiproteinase, antitrypsin), member 3 |
| SERPINE1 | serpin peptidase inhibitor, clade E (nexin, plasminogen activator inhibitor type 1), member 1 |
| SERPING1 | serpin peptidase inhibitor, clade G (C1 inhibitor), member 1, (angioedema, hereditary) |
| SGK | serum/glucocorticoid regulated kinase |
| SGNE1 | secretory granule, neuroendocrine protein 1 (7B2 protein) |
| SH3BP5 | SH3-domain binding protein 5 (BTK-associated) |
| SKIL | SKI-like |
| SLC25A24 | solute carrier family 25 (mitochondrial carrier; phosphate carrier), member 24 |
| SLC35D2 | solute carrier family 35, member D2 |
| SLCO1C1 | solute carrier organic anion transporter family, member 1C1 |
| SLCO2B1 | solute carrier organic anion transporter family, member 2B1 |
| SNAPC4 | small nuclear RNA activating complex, polypeptide 4, 190kDa |
| SNX10 | sorting nexin 10 |
| SOCS3 | suppressor of cytokine signaling 3 |
| SRPX2 | sushi-repeat-containing protein, X-linked 2 |
| STON1 | stonin 1 |
| SULT1A2 | sulfotransferase family, cytosolic, 1A, phenol-preferring, member 2 |
| SVIL | supervillin |
| SYNC1 | syncoilin, intermediate filament 1 |
| TAF13 | TAF13 RNA polymerase II, TATA box binding protein (TBP)-associated factor, 18kDa |
| TAGLN | transgelin |
| TES | testis derived transcript (3 LIM domains) |
| TGFB2 | transforming growth factor, beta 2 |
| TGFBI | transforming growth factor, beta-induced, 68kDa |
| THBS1 | thrombospondin 1 |
| TIMP3 | TIMP metallopeptidase inhibitor 3 (Sorsby fundus dystrophy, pseudoinflammatory) |
| TMEM158 | transmembrane protein 158 |
| TMEM63A | transmembrane protein 63A |
| TNFRSF25 | tumor necrosis factor receptor superfamily, member 25 |
| TPBG | trophoblast glycoprotein |
| TPK1 | thiamin pyrophosphokinase 1 |
| TPM2 | tropomyosin 2 (beta) |
| TRIP11 | thyroid hormone receptor interactor 11 |
| TSPYL5 | TSPY-like 5 |
| TTLL7 | tubulin tyrosine ligase-like family, member 7 |
| U2AF1L1 | U2(RNU2) small nuclear RNA auxillary factor 1-like 1 |
| USP36 | ubiquitin specific peptidase 36 |
| VCAM1 | vascular cell adhesion molecule 1 |
| WNT5A | wingless-type MMTV integration site family, member 5A |
| WWTR1 | WW domain containing transcription regulator 1 |
| XIST | X (inactive)-specific transcript |
| ZMYM5 | zinc finger, MYM-type 5 |
| ZNF250 | zinc finger protein 250 |
| ZNF365 | zinc finger protein 365 |
