# Supplementary material for: Astrocytes Enhance the Invasion Potential of Glioblastoma Stem-Like Cells
Source: PLoS One. 2013 Jan 22;8(1):e54752. doi: 10.1371/journal.pone.0054752 (PMC3551925; doi:10.1371/journal.pone.0054752)
Supplement: Table S3 — Genes (169) whose expression was commonly affected only after the direct co-culture of NSC11 and GBAM1 GSCs with astrocytes. Highlighted genes are present in the network shown in Fig. 5C. (DOCX) [file pone.0054752.s006.docx]

Table S3. Genes (169) whose expression was commonly affected only after the direct co-culture of NSC11 and GBAM1 GSCs with astrocytes. Highlighted genes are present in the network shown in Fig. 5C.

| ACTN2 | actinin, alpha 2 | |
| --- | --- | --- |
| **ADAM10** | **a disintegrin and metalloproteinase domain 10** | |
| ADAM9 | ADAM metallopeptidase domain 9 (meltrin gamma) | |
| ADFP | adipose differentiation-related protein | |
| ADORA2B | adenosine A2b receptor | |
| **AHR** | **aryl hydrocarbon receptor** | |
| AIM1 | absent in melanoma 1 | |
| **ALB** | **albumin** | |
| ALK | anaplastic lymphoma kinase (Ki-1) | |
| AMIGO2 | adhesion molecule with Ig-like domain 2 | |
| ANKRD26 | ankyrin repeat domain 26 | |
| ARID5A | AT rich interactive domain 5A (MRF1-like) | |
| ATHL1 | ATH1, acid trehalase-like 1 (yeast) | |
| **AXL** | **AXL receptor tyrosine kinase** | |
| **BIRC4** | **baculoviral IAP repeat-containing 4** | |
| BLVRB | biliverdin reductase B (flavin reductase (NADPH)) | |
| C14orf78 | chromosome 14 open reading frame 78 | |
| CALB1 | calbindin 1, 28kDa | |
| **CALD1** | **caldesmon 1** | |
| **CAV1** | **caveolin 1, caveolae protein, 22kDa** | |
| **CAV2** | **caveolin 2** | |
| **CDKN2A** | **cyclin-dependent kinase inhibitor 2A (melanoma, p16, inhibits CDK4)** | |
| CLIC2 | chloride intracellular channel 2 | |
| COL13A1 | collagen, type XIII, alpha 1 | |
| **COL18A1** | **collagen, type XVIII, alpha 1** | |
| **COL1A2** | **collagen, type I, alpha 2** | |
| **COL3A1** | **collagen, type III, alpha 1 (Ehlers-Danlos syndrome type IV, autosomal dominant)** | |
| COL6A1 | collagen, type VI, alpha 1 | |
| **CTGF** | **connective tissue growth factor** | |
| **CXCL12** | **chemokine (C-X-C motif) ligand 12 (stromal cell-derived factor 1)** | |
| **DACH1** | **dachshund homolog 1 (Drosophila)** | |
| DCAMKL2 | Doublecortin and CaM kinase-like 2 | |
| DCBLD2 | discoidin, CUB and LCCL domain containing 2 | |
| DDX58 | DEAD (Asp-Glu-Ala-Asp) box polypeptide 58 | |
| DENND4A | DENN/MADD domain containing 4A | |
| DIRAS3 | DIRAS family, GTP-binding RAS-like 3 | |
| **DKK1** | **dickkopf homolog 1 (Xenopus laevis)** | |
| DLG1 | discs, large homolog 1 (Drosophila) | |
| DNASE11 | deoxyribonuclease I-like 1 | |
| DYM | dymeclin | |
| EEA1 | early endosome antigen 1, 162kD | |
| **EGR2** | **early growth response 2 (Krox-20 homolog, Drosophila)** | |
| **F2RL1** | **coagulation factor II (thrombin) receptor-like 1** | |
| FABP5 | fatty acid binding protein 5 (psoriasis-associated) | |
| FAM134B | family with sequence similarity 134, member B | |
| FAM63B | family with sequence similarity 63, member B | |
| FBN2 | fibrillin 2 (congenital contractural arachnodactyly) | |
| FER1L3 | fer-1-like 3, myoferlin (C. elegans) | |
| **FLJ13615** | **hypothetical protein FLJ13615** | |
| FLJ21924 | hypothetical protein FLJ21924 | |
| FLNC | filamin C, gamma (actin binding protein 280) | |
| **FN1** | **fibronectin 1** | |
| **FOS** | **v-fos FBJ murine osteosarcoma viral oncogene homolog** | |
| GADD45A | growth arrest and DNA-damage-inducible, alpha | |
| GLIPR1 | GLI pathogenesis-related 1 | |
| GOLGA8B | golgi autoantigen, golgin subfamily a, 8B | |
| GPNMB | glycoprotein (transmembrane) nmb | |
| GPR51 | G protein-coupled receptor 51 | |
| HERC4 | hect domain and RLD 4 | |
| **HIST1H4E** | **histone 1, H4e** | |
| **HLA-B** | **major histocompatibility complex, class I, B** | |
| **HLA-E** | **major histocompatibility complex, class I, E** | |
| HRASLS3 | HRAS-like suppressor 3 | |
| HSPA2 | heat shock 70kDa protein 2 | |
| **ICAM1** | **intercellular adhesion molecule 1 (CD54), human rhinovirus receptor** | |
| IER3 | immediate early response 3 | |
| IF | I factor (complement) | |
| IFITM1 | interferon induced transmembrane protein 1 (9-27) | |
| IFITM3 | interferon induced transmembrane protein 3 (1-8U) | |
| **IGFBP7** | **insulin-like growth factor binding protein 7** | |
| **IL6ST** | **interleukin 6 signal transducer (gp130, oncostatin M receptor)** | |
| ISG20 | interferon stimulated exonuclease gene 20kDa | |
| **ITGB5** | **integrin, beta 5** | |
| **ITPR3** | **inositol 1,4,5-triphosphate receptor, type 3** | |
| KCNMA1 | potassium large conductance calcium-activated channel, subfamily M, alpha member 1 | |
| KIAA0367 | prune homolog 2 (Drosophila) | |
| **KIAA0690** | **ribosomal RNA processing 12 homolog (S. cerevisiae)** | |
| KIAA0830 | endonuclease domain containing 1 | |
| KIAA1164 | family with sequence similarity 63, member B | |
| **KNG** | **kininogen** | |
| **LAMB1** | **laminin, beta 1** | |
| LDLRAP1 | low density lipoprotein receptor adaptor protein 1 | |
| LEPREL2 | leprecan-like 2 | |
| **LIPG** | **lipase, endothelial** | |
| LOC283687 | hypothetical protein LOC283687 |  |
| LOC54103 | hypothetical protein LOC54103 |  |
| LOXL1 | lysyl oxidase-like 1 |  |
| **LOXL2** | **lysyl oxidase-like 2** |  |
| **LTBP2** | **latent transforming growth factor beta binding protein 2** |  |
| MALT1 | mucosa associated lymphoid tissue lymphoma translocation gene 1 |  |
| MAN2A1 | mannosidase, alpha, class 2A, member 1 |  |
| MAOB | monoamine oxidase B |  |
| MAP3K6 | mitogen-activated protein kinase kinase kinase 6 |  |
| **MAP4K5** | **mitogen-activated protein kinase kinase kinase kinase 5** |  |
| MDFIC | MyoD family inhibitor domain containing |  |
| **MDM2** | **Mdm2, transformed 3T3 cell double minute 2, p53 binding protein (mouse)** |  |
| ME1 | malic enzyme 1, NADP(+)-dependent, cytosolic |  |
| **MEG3** | **maternally expressed 3** |  |
| MFAP5 | microfibrillar associated protein 5 |  |
| MGC12262 | hypothetical protein MGC12262 |  |
| MICALL2 | MICAL-like 2 |  |
| MLPH | melanophilin |  |
| MVP | major vault protein |  |
| MXRA5 | matrix-remodelling associated 5 |  |
| MXRA8 | matrix-remodelling associated 8 |  |
| MYLK | myosin, light polypeptide kinase |  |
| MYO1B | myosin IB |  |
| NDN | necdin homolog (mouse) |  |
| NME5 | non-metastatic cells 5, protein expressed in (nucleoside-diphosphate kinase) |  |
| NOX4 | NADPH oxidase 4 |  |
| **NRCAM** | **neuronal cell adhesion molecule** |  |
| **NRP1** | **neuropilin 1** |  |
| **NRP2** | **neuropilin 2** |  |
| NT5DC3 | 5'-nucleotidase domain containing 3 |  |
| NT5E | 5'-nucleotidase, ecto (CD73) |  |
| ODZ4 | odz, odd Oz/ten-m homolog 4 (Drosophila) |  |
| OGFRL1 | opioid growth factor receptor-like 1 |  |
| **P4HA2** | **procollagen-proline, 2-oxoglutarate 4-dioxygenase (proline 4-hydroxylase), alpha polypeptide II** |  |
| PAR5 | Prader-Willi/Angelman syndrome-5 |  |
| PHF11 | PHD finger protein 11 |  |
| PLEKHA1 | pleckstrin homology domain containing, family A (phosphoinositide binding specific) member 1 |  |
| PLEKHA5 | pleckstrin homology domain containing, family A member 5 |  |
| **PLS3** | **plastin 3 (T isoform)** |  |
| POLD4 | polymerase (DNA-directed), delta 4 |  |
| POSTN | periostin, osteoblast specific factor |  |
| **PPFIBP1** | **PTPRF interacting protein, binding protein 1 (liprin beta 1)** |  |
| PPM2C | protein phosphatase 2C, magnesium-dependent, catalytic subunit |  |
| PPP4R2 | protein phosphatase 4, regulatory subunit 2 |  |
| RAB20 | RAB20, member RAS oncogene family |  |
| RARRES3 | retinoic acid receptor responder (tazarotene induced) 3 |  |
| **RBP1** | **retinol binding protein 1, cellular** |  |
| RFX3 | regulatory factor X, 3 (influences HLA class II expression) |  |
| RGS17 | regulator of G-protein signalling 17 |  |
| RGS2 | regulator of G-protein signalling 2, 24kDa |  |
| **RPS6KA1** | **ribosomal protein S6 kinase, 90kDa, polypeptide 1** |  |
| **RUNX1** | **runt-related transcription factor 1 (acute myeloid leukemia 1; aml1 oncogene)** |  |
| SEC24A | SEC24 related gene family, member A (S. cerevisiae) |  |
| **SEMA3C** | **sema domain, immunoglobulin domain (Ig), short basic domain, secreted, (semaphorin) 3C** |  |
| SGNE1 | secretory granule, neuroendocrine protein 1 (7B2 protein) |  |
| **SKIL** | **SKI-like** |  |
| SLC25A24 | solute carrier family 25 (mitochondrial carrier; phosphate carrier), member 24 |  |
| SLC35D2 | solute carrier family 35, member D2 |  |
| SLCO1C1 | solute carrier organic anion transporter family, member 1C1 |  |
| SLCO2B1 | solute carrier organic anion transporter family, member 2B1 |  |
| SNAPC4 | small nuclear RNA activating complex, polypeptide 4, 190kDa |  |
| SNX10 | sorting nexin 10 |  |
| SRPX2 | sushi-repeat-containing protein, X-linked 2 |  |
| STON1 | stonin 1 |  |
| SULT1A2 | sulfotransferase family, cytosolic, 1A, phenol-preferring, member 2 |  |
| SYNC1 | syncoilin, intermediate filament 1 |  |
| **TAGLN** | **transgelin** |  |
| TES | testis derived transcript (3 LIM domains) |  |
| **TGFB2** | **transforming growth factor, beta 2** |  |
| **TGFBI** | **transforming growth factor, beta-induced, 68kDa** |  |
| **TIMP3** | **TIMP metallopeptidase inhibitor 3 (Sorsby fundus dystrophy, pseudoinflammatory)** |  |
| TMEM63A | transmembrane protein 63A |  |
| TNFRSF25 | tumor necrosis factor receptor superfamily, member 25 |  |
| TPBG | trophoblast glycoprotein |  |
| **TPM2** | **tropomyosin 2 (beta)** |  |
| TRIP11 | thyroid hormone receptor interactor 11 |  |
| TSPYL5 | TSPY-like 5 |  |
| TTLL7 | tubulin tyrosine ligase-like family, member 7 |  |
| U2AF1L1 | U2(RNU2) small nuclear RNA auxillary factor 1-like 1 |  |
| USP36 | ubiquitin specific peptidase 36 |  |
| **VCAM1** | **vascular cell adhesion molecule 1** |  |
| **WNT5A** | **wingless-type MMTV integration site family, member 5A** |  |
| WWTR1 | WW domain containing transcription regulator 1 |  |
| **XIST** | **X (inactive)-specific transcript** |  |
| **ZMYM5** | **zinc finger, MYM-type 5** |  |
